# Design principles for cancer therapy guided by changes in complexity of protein-protein interaction networks

**DOI:** 10.1186/s13062-015-0058-5

**Published:** 2015-05-28

**Authors:** Sebastian Benzekry, Jack A. Tuszynski, Edward A. Rietman, Giannoula Lakka Klement

**Affiliations:** Inria team MC2, Institut de Mathématiques de Bordeaux, Bordeaux, France; UMR CNRS 5251, University of Bordeaux, 351 cours de la Libération, Talence, Cedex 33405 France; Department of Oncology, Faculty of Medicine & Dentistry, University of Alberta, 116 St and 85 Ave, Edmonton, AB T6G 2R3 Canada; Department of Physics, University of Alberta, 116 St and 85 Ave, Edmonton, AB T6G 2R3 Canada; Newman-Lakka Institute, Floating Hospital for Children at Tufts Medical Center, 75 Kneeland St, Boston, MA 02111 USA; Department of Pediatric Hematology Oncology, Floating Hospital for Children at Tufts Medical Center, 755 Washington St, Boston, MA 02116 USA; Newman Lakka Institute for Personalized Cancer Care, Rare Tumors and Vascular Anomalies Center, Chef, Academic & Research Affairs, Pediatric Hematology Oncology, Floating Hospital for Children at Tufts Medical Center, 800 Washington Street, Box 14, Boston, MA 02111 USA

**Keywords:** Topology, Persistent homology, Betti number, Cancer, Protein interaction networks

## Abstract

**Background:**

The ever-increasing expanse of online bioinformatics data is enabling new ways to, not only explore the visualization of these data, but also to apply novel mathematical methods to extract meaningful information for clinically relevant analysis of pathways and treatment decisions. One of the methods used for computing topological characteristics of a space at different spatial resolutions is persistent homology. This concept can also be applied to network theory, and more specifically to protein-protein interaction networks, where the number of rings in an individual cancer network represents a measure of complexity.

**Results:**

We observed a linear correlation of *R* = −0.55 between persistent homology and 5-year survival of patients with a variety of cancers. This relationship was used to predict the proteins within a protein-protein interaction network with the most impact on cancer progression. By re-computing the persistent homology after computationally removing an individual node (protein) from the protein-protein interaction network, we were able to evaluate whether such an inhibition would lead to improvement in patient survival. The power of this approach lied in its ability to identify the effects of inhibition of multiple proteins and in the ability to expose whether the effect of a single inhibition may be amplified by inhibition of other proteins. More importantly, we illustrate specific examples of persistent homology calculations, which correctly predict the survival benefit observed effects in clinical trials using inhibitors of the identified molecular target.

**Conclusions:**

We propose that computational approaches such as persistent homology may be used in the future for selection of molecular therapies in clinic. The technique uses a mathematical algorithm to evaluate the node (protein) whose inhibition has the highest potential to reduce network complexity. The greater the drop in persistent homology, the greater reduction in network complexity, and thus a larger potential for survival benefit. We hope that the use of advanced mathematics in medicine will provide timely information about the best drug combination for patients, and avoid the expense associated with an unsuccessful clinical trial, where drug(s) did not show a survival benefit.

**Reviewers:**

This article was reviewed by Nathan J. Bowen (nominated by I. King Jordan), Tomasz Lipniacki, and Merek Kimmel.

**Electronic supplementary material:**

The online version of this article (doi:10.1186/s13062-015-0058-5) contains supplementary material, which is available to authorized users.

## Background

Over the past 50 years a large amount of genomic, proteomic and pathway information has become available. While the information is detailed, it is very fragmented and correlations with available epidemiological data are slow to emerge. We have shown previously that the complexity of cancer protein-protein interaction (PPI) networks can be quantified by degree-entropy, a statistical metric, strongly correlated with cancer patient survival [1]. The concept was validated by similar findings by Takemoto, who has shown that modularity correlates with survival [2]. We now explore the use of new topological measures known as persistent homology [3] and cycle-basis [4] on protein-protein interaction (PPI) networks and their interpolation on survival.

The manuscript provides evidence that network complexity, as represented by persistent homology and cycle-basis, is not only correlated with survival rates of cancer patients (i.e. the more complex the network the worse the survival), but also that the removal of specific proteins from the network (i.e. surrogate of protein inhibition in clinic) can lead to a decrease in network complexity. Because complexity correlates with survival, changes in network complexity caused by removal of specific proteins from the protein-protein interaction networks can be used to calculate gains or losses in survival. We propose to use these mathematical predictions of improvements in survival for selections of protein stimulators/inhibitors to use with therapeutic intent. For example, we have found that while the elimination of a node from the PPI network lowered the degree-entropy [1], the change was too small to translate to an improvement in survival rate. However, the elimination of individual proteins from the PPI network and re-computing the persistent homology, as opposed to degree-entropy, enhanced the ability to predict the importance of specific proteins in the network and its potential effect on survival.

The manuscript explores the possibility that the evaluation of network topology using a persistent homology approach may lead to development of a predictive mathematical model for the optimization of treatment response to targeted therapy. Even more importantly, because more than one protein can be targeted at the same time, the approach can be used for development of combination therapies in cases where more than one oncogenic mutation is present in a single tumor.

At present, several approaches to analyzing networks are being explored. The use of conventional statistical mechanics measures of complex networks [5] is well established, and will not be reviewed here. We will, however, briefly review two statistical metrics: degree-entropy and betweenness-centrality. Degree-entropy has been described previously [1] and is computed from the entropy of the degree for each node. Specifically the relationship is given by:

$$ H=-{\displaystyle \sum_{k-1}^{N-1}p(k)}1\mathrm{n}\left[p,(k)\right] $$

where p(k) represents a probability distribution on the nodes of the network, *p*(*k*) = *N*_*k*_/*N* with N_k_ the number of nodes with degree k and N is the total number of nodes. Betweenness-centrality is a measure of the centrality of a node. Given a network graph G(E,V) consisting of nodes V and edges E, the betweenness-centrality c_B_ is a measure of the centrality of a node, v. Typically it is the sum of the fractions of shortest paths that pass through v and is given by:

$$ {c}_B={\displaystyle \sum_{s,\;t\in \kern0.22em V}\frac{\sigma \left(s,t\Big|v\right)}{\sigma \left(s,t\right)}} $$

where *σ*(*s*, *t*) is the number of shortest paths between two nodes (s,t) and *σ*(*s*, *t*|*v*) is the number of those paths passing through nodes other than v.

In addition to the above-discussed statistical measures, a well-known description of networks can be based on graph spectra [5–8]. Typically, one computes the eigenvalues of the adjacency matrix (or the Laplacian matrix) of the network graph. The spectral study is almost always associated with dynamical systems for investigating the stability of the network. One well-known result is that the multiplicity of zero eigenvalues of the graph Laplacian is equal to the number of components in the graph [9], and the number of components within the graph is equal to a topological measure known as Betti [10].

Before discussing our topological approaches we briefly mention an approach based on symmetry groups, because it may be considered analogous to our approach, especially the automorphism group [9]. A set of automorphisms of a network graph Aut(G) forms a group, and the cardinality (the number of elements) of the group |Aut(G)| is a measure of the complexity of the network graph. Typically, the automorphism group is decomposed, or factored, into a minimum set of symmetry groups. Given a graph G(V,E), an automorphism is a permutation acting on the vertices. Those permutations that can preserve the adjacency of vertices are called automorphisms. Formally, an automorphism g of a graph G, has the property that for two vertices u, and v, u_g_ is adjacent to v_g_ if and only if, v is adjacent to u (i.e. an edge exists between vertices u and v). These symmetry groups are sometimes called motifs and are genuine symmetry groups, unlike the motifs of Milo et al. [11]. The size of the automorphism group (the cardinality) correlates with cancer patient survival, but these symmetry groups are distinct from the topological measure we now discuss.

Our approach uses topology, i.e. cycle basis and persistent homology.

1. *Cycle-basis* is the simplest topological measure of a network. Paths in networks are sequences of vertices that are connected by edges [5, 12], and may or may not be self-intersecting. Paths that do not self-intersect, self-avoiding walks, are called geodesics and Hamiltonian paths. These self-avoiding paths are of interest as they represent the cycle-basis of a network. Each simple cycle C in a graph G has associated with it a vector indexed on the edge set, E(C). Each cycle forms an incident vector
  $$ {b}_i(C)=\kern1em \left\{\begin{array}{l}0,\kern1em  if\kern1em {e}_i\notin E(C)\\ {}\kern1em 1,\kern1em  if\kern1em {e}_i\in E(C)\end{array}\right.. $$
  These incident vectors form a set spanning a binary space *C*_*GF*(2)_(*G*) known as the cycle space of the graph. As long as the vectors are linearly independent, their incidence vectors are also linearly independent over the binary space, GF(2), and the independent set is called the cycle-basis [4, 13]. Two independent manuscripts by Berger and by Kavitha discuss an algorithm (implemented by Python-NetworkX) the theoretical basis of which was first described by Paton in 1969 [14].
2. *Persistent homology* is a more involved topological measure of networks [3, 10, 15] an abstract *n*-simplex {*v*_0_, ⋯, *v*_*n*_} of a set of *n* + 1 called vertices. Persistent homology can be geometrically represented as the convex hull of these *n* + 1 vertices in an *n*-dimensional Euclidian space. Simplices are generalizations in any dimension of convex polytopes such as points (0-simplices), segments (1-simplices), triangles (2-simplices) and tetrahedra (3-simplices). The faces of an *n*-simplex *σ* are all the (*n*-1)-simplices, which are subsets of *σ*. For example, a 3-simplex (tetrahedron) contains four 2-faces. A simplicial complex *K*. is a collection of simplices such that all the faces of each simplex are also in *K*. Given a simplicial complex *K* and a field (in our case $\frac{\mathrm{\mathbb{Z}}}{2\mathrm{\mathbb{Z}}}$), the space of k-chains *C*_*k*_ is defined as the abstract vector space generated by the k-simplices (that is, the vector space of all formal sums *λ*_1_*σ*_1_ + … + *λ*_*r*_*σ*_*r*_ with the ${\lambda}_i\in \frac{\mathrm{\mathbb{Z}}}{2\mathrm{\mathbb{Z}}}$ and the *σ*_*i*_’s being k-simplices of K). On these vector spaces the boundary operators ∂_*k*_ : *C*_*k*_ → *C*_*k* − 1_ are the sum of the faces (with the convention that points have empty faces). Notice that in our case, since the field is $\frac{\mathrm{\mathbb{Z}}}{2\mathrm{\mathbb{Z}}}$, we don’t need to define an orientation on simplices. Chains of dimension *k* that have null boundary (i.e. chain in *Z*_*k*_ := {*c* ∈ *C*_*k*_; ∂_*k*_(*c*) = 0}) are called cycles (*Z* for Zykel, cycle in German) and *k* -chains that are the boundary of a *k* + 1 -chain (i.e. chains in *B*_*k*_ := {*b* ∈ *C*_*k*_; ∃ *c* ∈ *C*_*k* + 1_, *b* = ∂_*k* + 1_(*c*)}) are called boundaries. Notice that for any *k* ≥ 1 we have ∂_*k*_ ∘ ∂_*k* + 1_ = 0 (i.e. *B*_*k*_ ⊂ *Z*_*k*_) since any boundary is a cycle. The *k* -th homology group *H*_*k*_ is defined by ${H}_k={Z}_k/{B}_k$ meaning that, starting from all the cycles, the ones that are boundaries are killed when taking the quotient. The dimension of the *k*-th homology group is called the *k*-th Betti number and is denoted *β*_*k*_, and the *k*-th Betti number records the number of *k*-dimensional holes. The 0-th Betti number is the number of connected components of the simplicial complex. Given a filtration *K*^1^ ⊂ ⋯ ⊂ *K*^*n*^ of embedded simplicial complexes (an ordering), persistent homology records homology classes persisting between two indices *i* and *j*, i.e. cycles that remain non-boundaries from i until at least *j* (these could be killed by a boundary appearing later on). The homology group of dimension *k* persisting from *i* to *j* is defined by ${H}_k^{i,j}={Z}_k^i/\left({Z}_k^i{\displaystyle \cap }{B}_k^j\right)$.

Its dimension is called the *k* -th Betti number persisting from *i* to *j* and is denoted *β*_*k*_^*i*,*j*^. In our analysis, only dimension 0 and 1 Betti numbers were found of interest and we will not consider here cases with *k* > 1.

Up to now, we have defined persistent homology for a simplicial complex filtration; however, protein-protein interaction networks do not appear as simplicial complexes but rather as graphs (a set of vertices and a set of edges). A simplicial complex can be built from a graph in the following way. Associated with a graph is a distance *d* defined by the length of the shortest path between two nodes (for our purpose, every edge is assumed to have length 1), and this endows the graph with a metric structure. The actual measure of persistent homology is computed as a Rips complex - *K*^*t*^ associated with this metric space, where parameter *t* is then defined by:

- Vertices are the points of the space (the nodes of the network)
- An edge {*v*, *w*} belongs to *K*^*t*^ if and only if *d*(*v*, *w*) ≤ *t*, *t* ≥ 0
- A higher dimensional simplex is in *K*^*t*^ if all its faces already belong to *K*^*t*^

For our purpose, we will limit ourselves to parameters t = (0,1,2,3), leading to Betti numbers *β*_1_^*i*,*j*^, 0 ≤ *i* ≤ *j* ≤ 3.

**Fig. 1:**
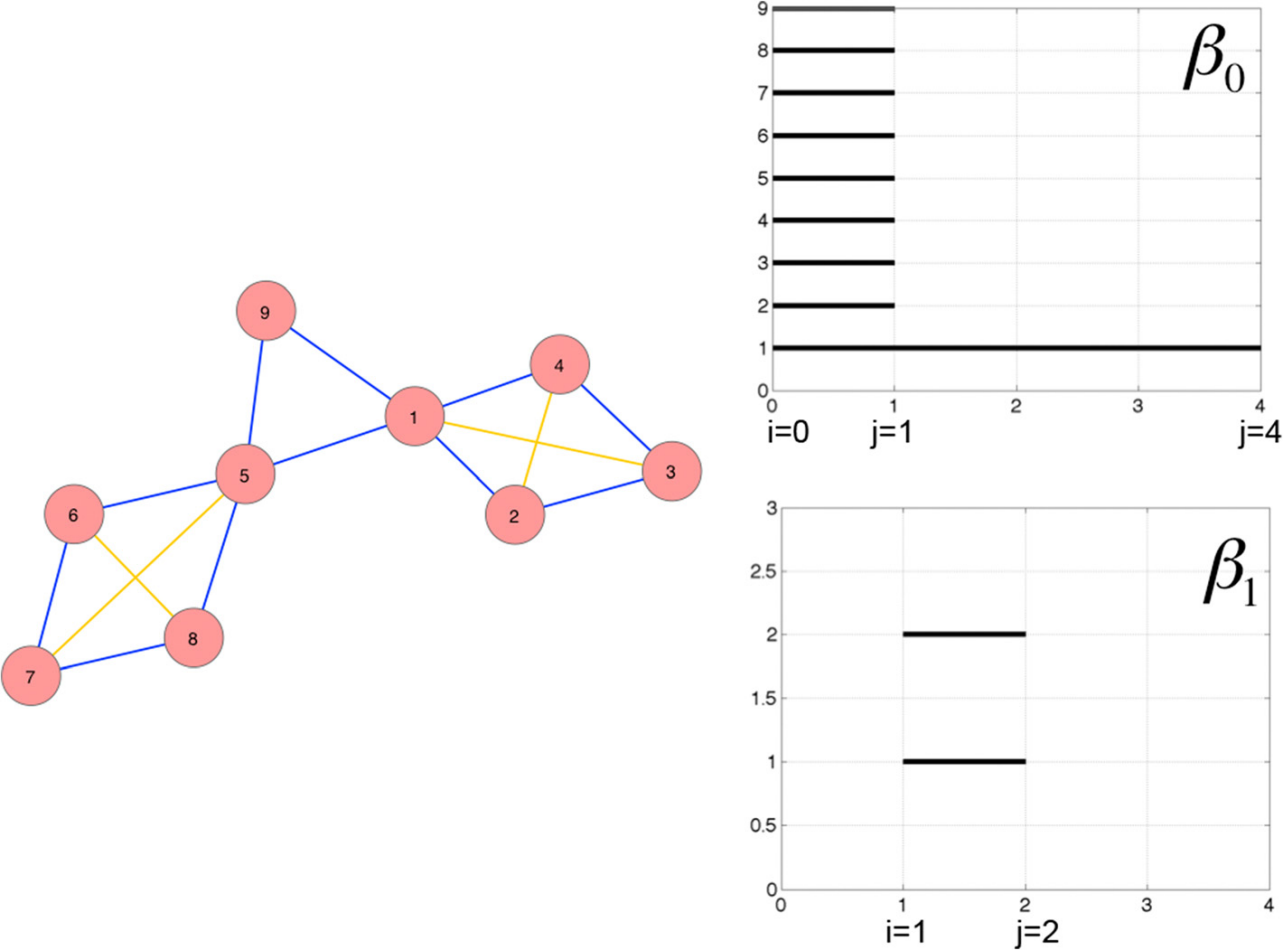
Example of Betti number calculation for a simple network. A simple network with red nodes and blue edges consists of two four-node cycles connected via a three-node cycle (Panel **a**). The Betti number algorithm adds the yellow edges in calculation of filtration index effectively “killing” the four-node cycles by eliminating the cycles. The numerical value for the Betti number is the number of bars lasting from index i to index j in a Betti barcode. Each bar starts when a homology class is born and ends when this class is killed due to appearance in the filtration of a higher dimensional chain whose boundary is the underlying cycle. Thus, Betti0 is equal to 9 and represents the number of nodes (Panel **b**), and Betti1, is equal to 2, and represents the number of rings of four nodes (Panel **c**)

To better understand what is encoded in the Betti numbers let us look first at a simple illustrative network presented in Fig. 1a. The red nodes and blue edges form the network, and the yellow edges are added as the filtration index increases. This simple network consists of two cycles of length 4 connected by a triangle (a cycle of length 3). The persistent homology (Betti numbers) of dimensions zero and one are shown in Fig. 1b and c, as so-called barcodes plotted against the filtration index (x-axis). A bar starts when a homology class is born and ends when this class is killed due to appearance in the filtration of a higher dimensional chain whose boundary is the underlying cycle. The measure for filtration index zero, Rips complex *K*^*0*^, the graph contains as one dimensional simplices the edges {*n*, *m*} such that *d*(*n*, *m*) ≤ 0 hence no edge at all and only the graph vertices (zero-dimensional simplices) are included. These nine vertices each account for one dimension in *Z*_0_^0^ and *B*_0_^0^ is empty. Thus, there is one homology class per vertex, explaining the nine bars lasting from 0 to 1 in Fig. 1b. These classes persist until index one, at which they get all killed but one. Indeed, *K*_1_ contains as one-dimensional simplices the edges {*n*, *m*} such that *d*(*n*, *m*) ≤ 1, that is all the edges of the graph (blue edges in Fig. 1a). Since the graph has only one connected component, every pair of two vertices is the boundary of a 1-chain and only one homology class remains in *H*_0_^0,*j*^ for all *j* ≥ 1. This shows that *β*_0_^0,*j*^ = 1 for *j* ≥ 1 and explains the only bar remaining after index 1 in Fig. 1b. These considerations also demonstrate that, for a general network, *β*_0_^0,*j*^ is the number of connected components of the network, for any *j* ≥ 1. The two distinct cycles of length 4 (namely *c*_1_ = {1, 2} + {2, 3} + {3, 4} + {4, 1} and *c*_2_ = {5, 6} + {6, 7} + {7, 8} + {8, 5}) present in the network generate two one-dimensional homology classes that appear at index 1 (Rips complex *K*^1^) but are not present in *K*^0^. There is also one cycle of length 3: *c*_3_ = {1, 5} + {5, 9} + {9, 1}. Each of these three cycles gives birth to one dimension in *Z*_1_^1^. However, only the two cycles of length 4 *c*_1_ and *c*_2_ remain as homology classes in *H*_1_^1^ because *c*_3_ is the boundary of the two-dimensional simplex {1, 5, 9} that belongs to *K*^1^. (because all its faces are elements of *K*^1^. . Hence *c*_3_ belongs to *B*_1_^1^ and kills the homology class created by {1, 5, 9} in *Z*_1_^1^. At index one we thus have *β*_1_^1,1^ = 2, explaining the two bars in Fig. 1c. They disappear at index two because *c*_1_ and *c*_2_ become boundaries. In *K*^2^, four edges are added to the ones already present in *K*^1^, namely {1, 3}, {2, 4}, {5, 7} and {6, 8} because they are between vertices {*v*}, {*w*} that are such that *d*(*v*, *w*) ≤ 2. These new edges (the yellow edges in Fig. 1) give birth to new two-dimensional simplices such as {1,2,3} and {1,3,4}. These fill the holes created by cycles *c*_1_ and *c*_2_ and consequently kill the two associated homology classes. It explains why *β*_1_^1,*j*^ = 0, for all *j* ≥ 2 and why the two bars in Fig. 1c last from index 1 to 2. This example shows that essential *β*_1_^1,1^ records the number of independent cycles of length 4 or more in the network. Cycles of length 4, 5 or 6 are killed when distance between two edges appear in the filtration and hence only last from index one to two. Larger cycles give longer-lasting one-dimensional homology. For instance a cycle of length 7 would give a homology class lasting from index 1 to 3. These simple examples of networks with one-dimensional cycles do not exhibit homology (holes) of dimension higher than one. Such features are obtained with slightly more complex graphs such as the one that would be obtained from the graph of an octahedron and its edges (one two- dimensional homology class created at index 1 and lasting until index 2).

In summary, the homology measures we defined, persistent homology/ Betti numbers are measures of (i) the number of independent *n*-dimensional cycles, and (ii) the amount of connectivity that would be needed in order to annihilate these cycles. These homology measures are therefore sensitive metrics of the network complexity and, depending on the node location and its connectivity, its removal from the network may have significant or insignificant effect.

## Results and discussion

**Fig. 2:**
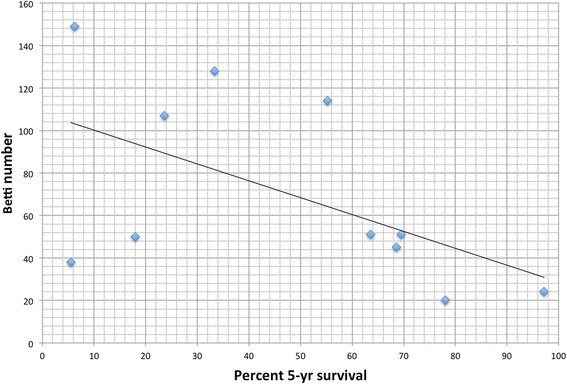
Mapping of Betti numbers onto survival data from Surveillance Epidemiology and End Results (SEER) database. Nominal Betti numbers were calculated on the Kyoto Encyclopedia of Genes and Genomes (KEGG) and correlated with Surveillance, Epidemiology and End Results Database (SEER). Nominal Betti, a measure of network complexity, was inversely correlated with percent 5-year survival (*R* = −0.551, *p* = 0.0789), suggesting an inverse relationship between network complexity and cancer survival

We computed the Betti number for each cancer network and plotted that value versus the five-year survival rate. This is shown in Fig. 2. The R correlation is −0.55 and the p-value is 0.079. We systematically removed each node in each network and recorded the change in Betti number. The single node protein removal that resulted in the largest drop in Betti number also resulted in the largest drop in complexity, and as we discuss below, is potentially a good drug target. These results are shown in Table 1. We also systematically removed two proteins from the network and recomputed the Betti number to observe which double inhibition would lead to the lowest Betti number. These results are shown in Table 2.

**Table 1:** Betti number analysis and its implication for single protein targeting. The Surveillance, Epidemiology and End Results Database (SEER) provide data for the 5-year survival, degree-entropy was calculated from the Protein-protein interaction network (PPI) using Python®, Cycle-Basis was calculated using networkX.cycle_basis (a Python function), the nominal Betti number was calculated using JPLEX, and represents the Betti number for the full cancer network, and the Best Betti represents the lowest Betti number achieved by removal of a protein from the specific cancer PPI network. Degree-entropy and cycle basis are provided only as alternative measures of network complexity. When the elimination of proteins generated the same reduction in Betti, they were considered “equivalent targets”. A node connecting two clusters of proteins would be considered a node with high betweeness. We used highest betweeneness-centrality to differentiate equivalency targets, and the shaded entries have lowest Betti numbers and high betweeness centrality in the specific cancer

		AML	Bladder	CML	Colorectal	Endometrial	Glioma	NSCL	Pancreatic	Renal	SCL	Thyroid
5 years Survival [%]		23.6	78.1	55.2	63.6	68.6	33.4	18	5.5	69.5	6.2	97.2
Degree-Entropy		2.16	1.52	2.11	1.63	1.6	2.22	2.23	2	1.59	2.06	1.38
Cycle-Basis		108	20	115	58	45	128	75	72	52	150	24
Nominal Betti		107	20	114	51	45	128	50	38	51	149	24
Best Betti		95	15	101	41	35	109	37	29	34	131	17
Equivalent Targets		FLT3									ITGA3	
		HRAS	MAPK3	AKT1	AKT3		HRAS				ITGA6	HRAS
		NRAS	MAPK1	AKT2	AKT2	PDPK1	NRAS	KRAS	KRAS	HIF1A	ITGA2B	NRAS
		KRAS		AKT3	AKT1	ILK	KRAS			EPAS1	ITGB1	KRAS
											ITGA2	
											ITGAV

**Table 2:** Betti number analysis and its implication for multiple protein targeting. The data for the 5-year survival was again derived from Surveillance, Epidemiology and End Results Database (SEER), the nominal Betti number was calculated using JPLEX, and represents the Betti number for the full cancer network, and the Best Betti represents the lowest Betti number achieved by removal of a protein from the specific cancer protein-protein interaction (PPI) network. Through sequential removal of protein pairs across the entire PPI network of each cancer, we were able to identify combinations of protein pairs exhibiting most effect on the PPI network. Shown are the node combinations which produce the most reduction in network complexity as measured by the “Best Betti, double node elimination”, each paired node elimination results in equivalent drop in Betti, e.g. HRAS-FLT3 and HRAS-NRAS both have Best Betti of 83 in AML

		AML	Bladder	CML	Colorectal	Endometrial	Glioma	NSCL	Pancreatic	Renal	SCL	Thyroid
5 years Survival [%]		23.6	78.1	55.2	63.6	68.6	33.4	18	5.5	69.5	6.2	97.2
Nominal Betti		107	20	114	51	45	128	50	38	51	149	24
Best Betti [single node elimination]		95	15	101	41	35	109	37	29	34	131	17
Best Betti [double node elimination]		83	11	88	31	26	90	29	21	26	113	10
Paired node elimination			HRAS – MAPK3								ITGA2 – ITGA3	
			ARAF – MAPK3								ITGA2 – ITGA6	
			RAF1 – MAPK3								ITGA3 – ITGA6	
		HRAS – FLT3	NRAS – MAPK3								ITGA2 – ITGA2B	
		HRAS – NRAS	HRAS – MAPK1	AKT1 – AKT2	AKT1 – AKT2		NRAS – HRAS				ITGA3 – ITGA2B	HRAS – NRAS
		FLT3 – NRAS	ARAK – MAPK1	AKT1 – AKT3	AKT1 – AKT3	ILK – PDPK1	NRAS – KRAS	EGFR – KRAS	NFKB1 – KRAS	HIF1A – GAB1	ITGA6 – ITGA2B	HRAS – KRAS
		HRAS – KRAS	RAF1 – MAPK1	AKT2 – AKT3	AKT2 – AKT3		HRAS – KRAS	ERBB2 – KRAS	RELA – KRAS	GAB1 – EPAS1	ITGA2 – ITGB1	NRAS – KRAS
		FLT3 – KRAS	NRAS – MAPK1								ITGA3 – ITGB1	
		NRAS – KRAS	MAPK3 – KRAS								ITGA6 – ITGB1	
			MAPK1 – KRAS								ITGA2B – ITGB1	
			MAPK3 – BRAF								ITGA2 – ITGAV	
			MAPK1 - BRAF								ITGA3 - ITGAV	

### Overview of results

Betti numbers and cycle-basis are used to describe a measure of complexity of the PPI networks within the cancer pathway networks. The Betti number is a measure of the number of the cycles with four or more nodes, whereas cycle-basis is the number of cycles with three or more nodes. Our analysis of persistent homology of cancer networks revealed that a particular Betti number, symbolized as *β*_1_^1,1^, was highly correlated with survival. This particular Betti number represents the number of one-dimensional homology classes of the network. We focus only on this number in the subsequent text, and simply refer to it as the Betti number associated with the network. This Betti number is linearly correlated (*R* = 0.932) with cycle-basis. In general, a Betti number represents a topological measure – an abstraction – of the network. The abstraction of the network into Betti numbers facilitates an analysis of the correlation between Betti numbers and percent survival. The mapping the Betti numbers space onto the survival curves using a computational algorithm, indirectly maps the PPI-space onto survival space. This indirect mapping is of central interest because we are abstracting a large amount of network information into a single number, and this abstraction is associated with a particular amount of uncertainty. Thus, the uncertainty of mapping Betti number-space to percent survival-space leads to a significant increase in uncertainty. Because many networks can give rise to the same Betti number (surjective mapping), Betti number mapping to survival must be done with this caveat in mind.

The actual mapping of Betti numbers onto the Surveillance Epidemiology and End Results (SEER) database (Fig. 2) revealed an inverse linear correlation (*R* = −0.551, p-value 0.079) between Betti number (a dimension of persistent homology from filtration index one to two) and percent 5-year survival for a variety of cancers. The inverse correlation implies that the higher the Betti number, the lower the probability of five-year survival. Our interest in this approach lies not simply in describing this mapping, but rather in using such a mapping for developing tools for evaluation of therapeutic targets and designing personalized therapies.

A similar correlation was reported previously for the relationship between the degree-entropy and survival mapping [1], where the authors concluded that a targeted removal of a single protein from the network (the clinical equivalent of this would be a pharmacological inhibition of the protein) resulted in very little change in the degree-entropy unless the protein was a key hub (a protein with large number of connections to other proteins). Given the negative correlation of survival and Betti numbers in our framework, the elimination of a protein from the network leads to a decrease in Betti number. The degree of Betti number change corresponded to the importance of the protein in the network. Consequently, when the elimination of a protein from the network leads to a much lower Betti number, the protein represents a potential therapeutic target. In contrast, the elimination of proteins in the periphery of the network (i.e. leaf nodes that have only one connection) has little or no impact on Betti number, degree-entropy, or cycle-basis. In contrast, the targeted elimination of hub proteins (those with a large number of connections) can lead to collapse of the network, and such a target may not be the best to be explored therapeutically. Potential therapeutic targets with high impact on the network can be investigated as sets of proteins. Even though the elimination of proteins internal to the network can lead to small changes in degree-entropy [1], it had a significant impact on the Betti number, suggesting that the use of Betti numbers in evaluation of network complexity is superior to degree-entropy.

### Discussion of Table 1

The ability to use persistent homology and complexity of cancer PPI networks for the exploration of the importance of specific cancer-related proteins has a great potential for distinguishing “driver” mutations from those that represent “passenger” events. Because Betti numbers for networks are sensitive to individual as well as to elimination of multiple protein inhibitions, they can be used for defining combinatorial therapies. An analysis of the effect(s) of inhibiting a single node (Table 1) or multiple nodes (Table 2), the respective Betti numbers and percent 5-year survival, provides implications for drug targeting. The “nominal Betti number” is the Betti number calculated from the intact KEGG/PPI cancer network. The “best Betti number” is the lowest Betti number obtained by removal of a protein from the specific cancer PPI network. We include in Table 1 degree-entropy and cycle basis as alternative measures of network complexity for comparison. While in present clinical practice it is accepted to treat any change in protein expression (compared to normal tissues) as equivalent in importance, the findings in Table 1 suggest otherwise. Some of the proteins have a significant effect on the integrity of the network while others come along for the ride. For example, the ras family of proteins is very important in glioma, and removing one of HRAS, KRAS or NRAS leads to a significant decrease in network complexity, reflected in 15 % reduction in Betti number, a decrease from 128 to 109 (Table 1). In comparison, the role of the ras family of proteins is less important in chronic myeloid leukemia or endometrial cancers. In these cancers, removing any single ras family member had little impact on the complexity of the respective cancer networks, yielding only 4 % and 9 % Betti number reductions (from 114 to 110 and from 45 to 41 respectively, data not shown). Similarly, in non-small cell lung cancer (NSCLC), and in pancreatic cancer, KRAS appears to be one of the main drivers, and its targeting leads to a drop in Betti number from 50 to 37 (26 % reduction) for NSCL, and a drop from 38 to 29 (24 % reduction) for pancreatic cancer (Table 1).

### Discussion of Table 2

Betti numbers may therefore provide invaluable information in choosing combinatorial therapies. For example, targeting KRAS and EGFR together (or equivalently KRAS and ErbB2) leads, in the case of NSCL, to a synergistic reduction in complexity, and Betti number drops further from 37 to 29 (Table 2). Similarly, in acute myeloblastic leukemia (AML), a very hard to treat cancer with survival between 20 and 30 % for some of its subtypes, FLT3, HRAS, NRAS and KRAS mutations appear to have an equivalent effect in their ability to suppress the complexity of the cancer network (Betti number drops from 107 to 95). Moreover, data summarized in Table 2 suggests that a combination of two targets may lead to further enhancement of the effect. The elimination of both RAS and FLT3 for example, leads to a drop in Betti number to 83.

Cancer networks represent a very complex and highly interactive space. Reducing this complex information to a representative Betti number may facilitate clinically meaningful predictions of treatment response. In [1] we published a method of measuring complexity based on high betweenness-centrality. The term describes the frequency of short connections between two nodes, and implies that breaking these connections can lead to destruction of the network connectivity. However, the decrease of betweenness-centrality in this earlier publication did not always translate into a decrease in complexity as measured by degree-entropy. Combining information about high betweenness-centrality and Betti number (see shaded areas in Table 1) led to meaningful improvement in the accuracy of predicting clinical response. For example, in endometrial cancer, a PDPKI protein has both the highest betweenness-centrality (shaded in Table 1) and the highest drop in Betti number, suggesting it may be a very strong candidate for drug development.

### Detailed examples

**Fig. 3:**
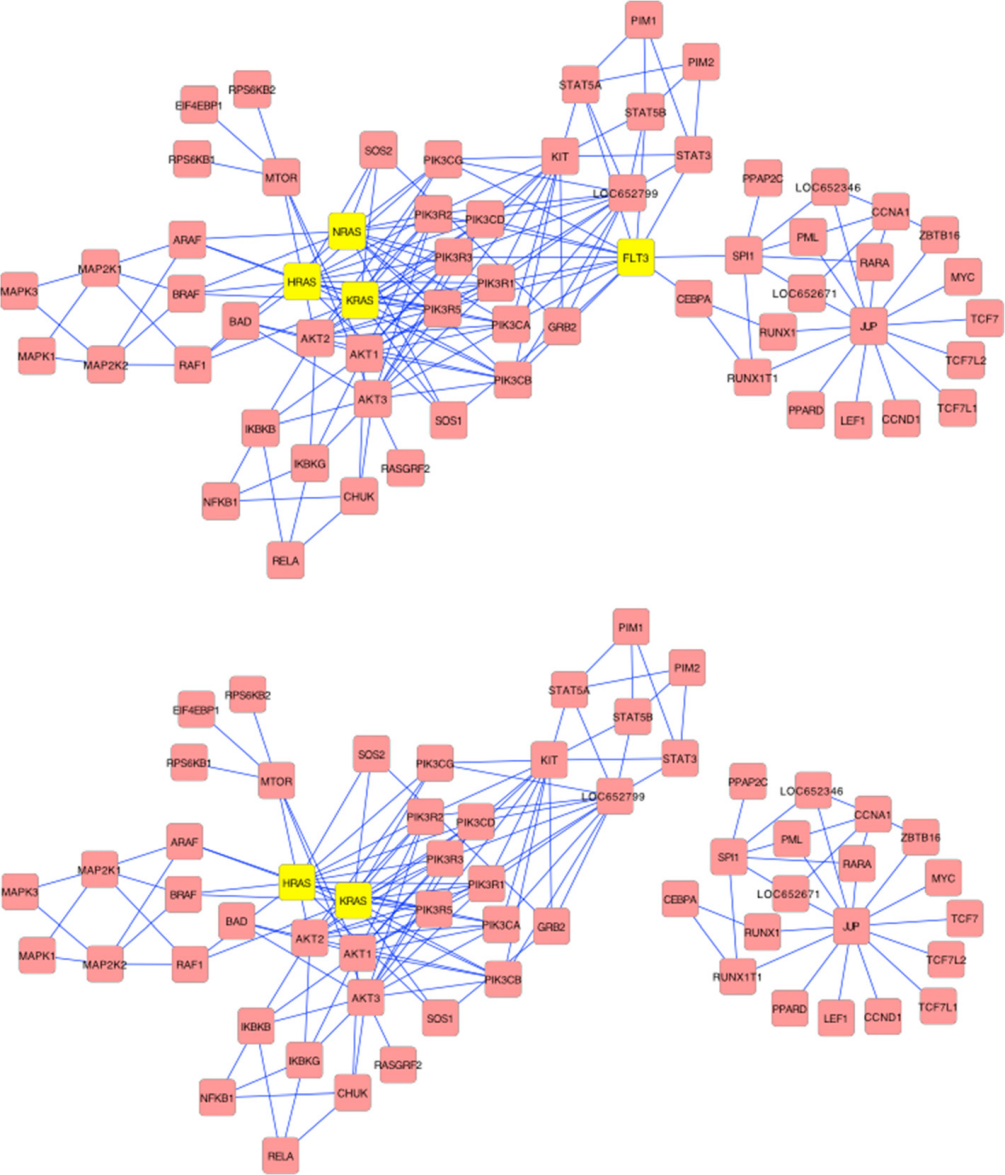
Analysis of the Protein-Protein Interaction network for Acute Myeloid Leukemia. Protein-protein interaction network (PPI) for AML was obtained from Kyoto Encyclopedia of Genes and Genomes (KEGG) and converted into the figure present in Panel **a** using R-script known as KEGGraph. It reveals a complex single component network, which breaks into two separate components by targeted elimination of the connecting FLT3 protein. The effect of simultaneous elimination of NRAS and FLT3 (Panel **b**) leads not only to break down of the network into two separate components, but also to thinning of the main network

**Fig. 4:**
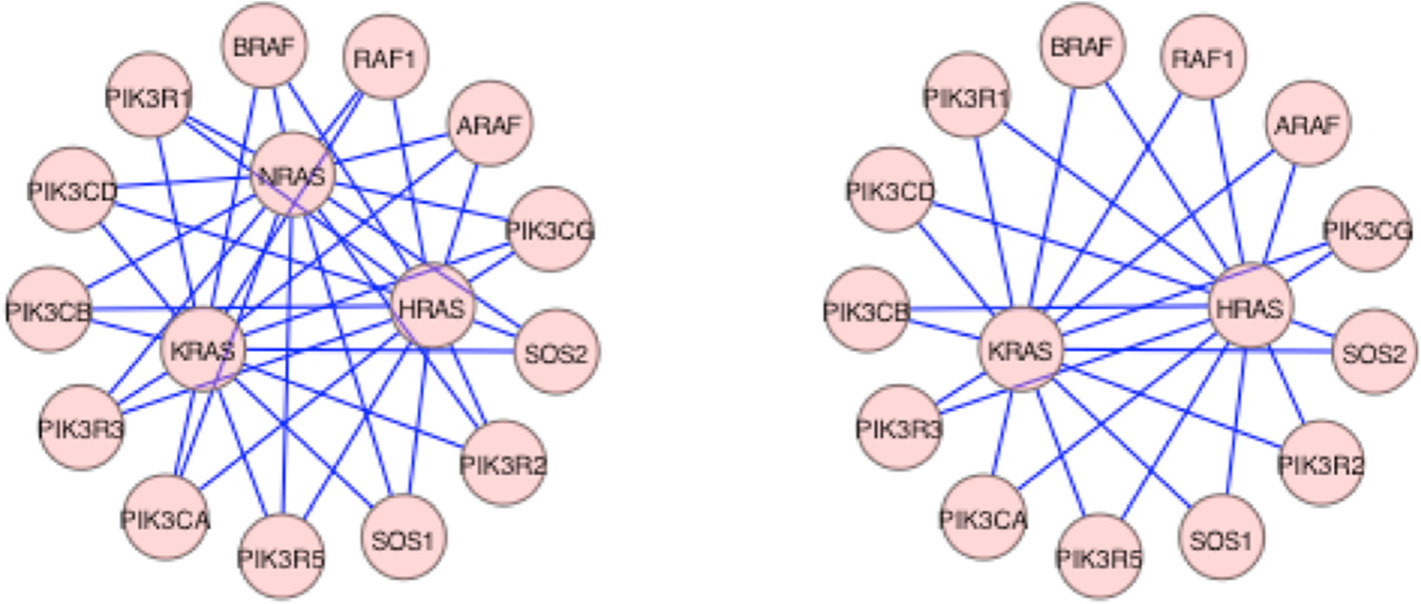
Effect of elimination of more than one Betti-derived targets from the Acute Myeloid Leukemia Protein-Protein Interaction network. The AML PPI network was imported into Cytoscape®, and all nodes except for the RAS family and their neighbors, were eliminated for graphical purposes only. As seen in panel **a**, the RAS family of proteins indicates that each of respective proteins (HRAS, KRAS and NRAS) is equivalent in importance and interconnected with similar neighbors. Panel **b** shows the effect of inhibition of NRAS and the resulting reduction in complexity

Thus, to understand how Betti number could translate to development of biologically- based therapies, we start with the elimination (inhibition) of a specific protein within a particular network. The protein-protein interaction (PPI) network for AML based on KEGGgraph (Fig. 3a) graphically depicts the best targets determined by Betti number. FLT3, which has high betweenness-centrality can be seen to connect two sub-neworks. The beneficial effects of inhibiting NRAS and FLT3 simultaneously is evident in Fig. 3b. The network breaks into two “components” but the inhibition does not result in a complete destruction of the network, which would have been potentially lethal. The details of the ras family of proteins effect are depicted in Fig. 4. NRAS, KRAS, and HRAS, appear to be completely equivalent in their linkages, suggesting biological redundancy (see Fig. 4b where NRAS and its linkages have been removed). The topology shows that any of the RAS family proteins are very good targets in AML, bladder, glioma and thyroid (Table 2). However, in NSCL and pancreatic cancer only KRAS plays a significant role (Table 1). This is consistent with the present understanding in the clinical community [16].

Note that the elimination of FLT3 and any one of the RAS proteins leads to break down of the network into two separate components as well as thinning of the major network, suggesting that the combination of agents that inhibit both subnetworks would be synergistic. This double protein inhibition resulted in greater reduction in Betti number from 107 to 83 (Table 2). This reduction is again strongly correlated with survival. While complete destruction of the network appears to be possible by targeting all three RAS proteins along with the FLT3, such an approach is suggestive of significant toxicities as it collapses all physiological pathways.

## Conclusions

In summary, our work suggests that Betti numbers may represent a sensitive measure of network complexity and may be useful for optimizing the design of protein-protein interactions within networks. Because the reduction of network complexity achieved by elimination of one or more proteins from the PPI network can be correlated with changes in survival rates, the method holds potential for optimization of targets for personalized targeted therapies. The most reassuring finding, a post-diction, was that the mathematical derivation of Betti number calculations, “discovered” a number of drug targets successfully used for treatment in clinics and performing well in clinical trials. Similarly, clinical trials using targets whose elimination did not lead to a significant drop of Betti number were not successful. It is likely that the use of mathematics in medicine would decrease the frequency of negative clinical trials as well as provide timely information about the best drug combination for individual patients.

## Methods

For most cancers a great deal of molecular information is now available. The information has been for many cases compiled, curated, and posted at the Kyoto Encyclopedia of Genes and Genomes (KEGG) [17], and is publicly available at http://www.genome.jp/kegg/pathway.html. Similarly, detailed statistical information about the five-year survival rates of patients with cancer is readily available at the Surveillance Epidemiology and End Results (SEER) National Cancer Institute database. We have previously published the details of how the KEGG pathway data can be converted into protein-protein interaction networks [1]. Briefly, the approach involves processing the KGML files representing the pathway network using a software program known as KEGGgraph [18] available at Bioconductor (http://www.bioconductor.org/packages/2.4/bioc/html/KEGGgraph.html). The R-script converts the KEGG file into an adjacency list for a protein-protein interaction (PPI) network, and using DAVID [19] (http://david.abcc.ncifcrf.gov/) one can convert the Entrez IDs into gene symbols. Cytoscape [20] can then be used for visualization and analysis of the PPI networks. The betweenness-centrality was calculated using the Python code networkx.betweenness_centrality(G), and the betweenness-centrality values were confirmed with a Cytoscape Network Analysis plugin.

To compute the persistent homology/ Betti numbers associated with the cancer networks, we used the Matlab implementation of a software package for JPlex (http://www.swmath.org/software/9851).

## Reviewers’ comment

### Reviewer 1: Dr. Nathan J. Bowen (nominated by Dr. I. King Jordan): Clark Atlanta University, United States of America

While the formal mathematical work is sound, the lack of measurements of the actual protein levels (nodes) precludes any biological insight that can be gained by measuring the topology of the network. Just because a protein is listed in the signaling network, it doesn’t mean that it is present in the diseased state. Many proteins (nodes) that may represent potential tumor suppressors in each of the pathways presented are often lost in cancer or their expression level is severely reduced. Cancer KEGG pathways draw from what is known about the circuitry of signaling pathways that have been deduced from the study of multicellular embryonic development as well as cancer.

In the case of embryonic development, the pathways are meant to include the necessary components for “wild type” development, that is, functional pathways that lead to “healthy” cellular homeostasis. Cancer is considered a genetic disease. Mutations that lead to loss of proteins (tumor suppressor nodes) or gain or overexpression of proteins (oncogenic nodes) are detected in virtually all cancers analyzed. Therefore, some measure of expression level, or present/ absent value must be assigned to each node in a healthy state vs. a cancer state before differential topological calculations can produce meaningful biological insights that translate into clinical decision making at the treatment and progression levels.

In a publication by West J, Bianconi G, Severini S, Teschendorff AE. Differential network entropy reveals cancer system hallmarks. Scientific Reports 2012;2:802. doi:10.1038/srep00802 the authors actually find that “the average of the absolute correlations over neighbours of a given node provides an equally good discriminator (Fig. S4), indicating that the loss of local connectivity is a key cancer characteristic.”

We also see a similar loss of connectivity in networks that we construct using gene expression data, that is, a reduction in the number of correlated gene pairs in cancer cell gene expression networks when compared to healthy cell gene expression networks.

The measure of persistent homolog is a highly plausible description of signaling network topology in biological systems. It is also a highly attractive measure to use when calculating differences between networks when nodes are removed as the authors have described here. When additional parameters are established for node quantification, persistent homology will surely be incorporated in experimental manipulation of node knock-out in living cells as is now routinely accomplished using CRISPR/Cas9 gene editing technology.

However, until quantities or at least presence/absence values are given to nodes in healthy vs. cancer states, I don’t feel that it is a reliable measure that can be used in the biological, much less clinical, interpretation of signaling or protein-protein interaction networks.

#### Author response to reviewer number 1

We agree with the reviewer that just because a protein is present in the KEGG signaling network for a specific cancer, does not mean it is differentially expressed between cancer and healthy tissues. We need to stress here that no information about gene expression is explicitly embedded in the KEGG networks, and as such it is not considered in our calculation of Betti numbers. The Betti number, as a measure of complexity of a protein-protein interaction network, simply reflects the number of cycles of four or more connected nodes. There is also no embryonic pathway information embedded in the cancer KEGG pathway networks. The KEGG pathways, not just cancer pathways, are constructed and curated by research staff at the Kyoto University, who read literature and manually build-in the connections. For example, a cancer such as glioblastoma will have its pathway built as an *average* pathway for that cancer. It does not and cannot include mutation information, or specific patient or cancer stage information. We used the KEGG networks in order to exploit protein-protein interactions for each cancer, and to analyze the topology of those networks. While our long-term goal is finding a technique to assist clinicians in their decision making, this manuscript presents a method of measuring PPI network complexity and provides some simple examples of how this method can, independently of expression data, point to those genes that are of importance. There is ongoing work to merge this topology measures with expression data and refine cancer specific approaches.

This also explains why the research report of West, et al. is not relevant. The report combines a fixed PPI network architecture with mRNA expression data to derive uniquely weighted networks for each of cancers they studied. Their weighted networks have fixed architecture across all cancers. Our architecture of the PPI network is NOT fixed. Our analysis is strictly based on topology of these unique networks, making Dr. West’s method singularly different from ours. One may view the two methods as potentially complementary methods for drug target selection. Dr. West’s team found that local entropy is a key factor in determining potential targets, and they were able to deduce important information about robustness of a particular node within the network. Their target suggestions are based mainly on mRNA expression levels across a population of samples. A protein with a very highly up-regulated mRNA expression is assumed to be of importance in the network – they do not actually compute network entropy. This is how many targets are presently “discovered”, by assuming that the strength of up- or down-regulation of a gene reflects its importance.

Our method, in contrast, analyzes each of the cancer PPI network *as a unique network* and avoids biasing its complexity with expression data. The shortfall of course is that the method relies on the quality of the KEGG PPI networks. However, it is extremely reassuring that despite the uncertainty associated with KEGG networks information, there is a strong correlation between network complexity and survival data from SEER.

### Reviewer 2: Dr. Tomasz Lipniacki Rice University, United States of America

Comments on Design Principles for Cancer Therapy guided by changes in complexity of Protein-Protein Interaction Networks by Benzekry et al. The Authors introduce topological measures of protein-protein interaction networks, such as Betti numbers (related to the structure of cycles in the interaction graph). Next, they analyze 11 cancer interaction networks to found that Betti number #11,1 (which measures the number of independent cycles of length larger than 3) correlates (negatively) with 5-year survival rate (Fig. 2). After that, they look into nodes, which removal lead to the highest decrease of Betti numbers, and also found pairs of such nodes. They propose that such nodes are good candidates for cancer therapy, as removing such nodes would substantially reduce complexity of network topology.

The presented results are interesting and in a sense intuitive. Larger Betti numbers correlate with networks connectivity and therefore its robustness to perturbation, which may imply stronger persistent of cancer network to therapy.

I think however, that the conclusion in which authors state that nodes which removal lead to the greatest reduction of network complexity goes a bit too far. First, cancer networks consist of proteins that have prosurvival (from cell perspective) and proapoptotic roles. Removing highly connected nodes with proapoptotic “action” would even worsen the prognosis. Second, nodes important for cancer network complexity, are likely to be important in non-cancerous cells, and therefore therapy which aims in blocking these nodes may have lethal sides effects. The Authors should discuss these points.

I think also that the statistics of 11 considered networks is too small to conclude (prove) that Betti number #11,1 is the best topological measure (i.e. with the strongest correlation). Analyzing Fig. 2, one can expect that removing just two points (say with the highest 5-years survival) would reduce correlation close to zero. One can thus think that having only 11 networks in analysis, it is possible to choose such a topological measure of the networks that would negatively (or positively) correlate with survival prognosis, even if there is no real correlation.

The credibility of study would be increased by analysis of the substantially larger number of cancer networks, or maybe other (non-cancer) networks for which the similar reasoning is possible, to show that Betti number #11,1 is a good measure of network robustness.

#### Authors response to reviewer number 2

We agree with the reviewer that the conclusions we draw about utility of Betti numbers as a measure of complexity may not be on its own sufficient for target selection. We simply imply that analysis of network complexity may initiate development of mathematical tools that may lead to a more rational selection of targets. We also agree with the reviewer that many other network measures should be and were explored in evaluating the degree of correlation between complexity of cancer PPI networks and five-year survival. For example, in addition to Betti numbers, we examined the following: degree entropy, number of leaf nodes, leafs per node, leafs per edge, leafs per component, average and standard deviation of degree, closeness centrality, clique, transitivity, assortivity, node count, edge count, number of connected components, the first five eigenvalues of the adjacency matrix, and the first five eigenvalues of the Lapalcian matrix. We only found three useful measures: Betti number, number of leafs, and degree entropy. We published a manuscript describing the correlation between degree entropy and survival (PMID: 22615392, Proc Natl Acad Sci U S A. 2012 Jun 5;109(23):9209-12). However, we found that computing the degree entropy after removing a node from the network led to a very small change in entropy, and the interpolation of this value on an entropy/survival curve was insignificant. We have not as of yet looked at whether the number of leafs as a metric may be useful. It was our goal to find a network metric, which would show a *linear* correlation with survival. In Betti number we found a metric with sufficiently robust change following removal of a node, that its interpolation on the linear Betti/survival curve could be predictive of a meaningful change in survival.

We also agree with the reviewer that many of the nodes important for cancer network complexity, are likely to be important in non-cancerous cells. This is a concern for most medical therapies and cannot be underestimated. However, there are many steps between the identification of a potential target and development of an agent, and for pathways that are vital to life we often chose a downstream inhibition, one that manages the symptoms rather than directly inhibits a node. A good example is the KRAS pathway, it is vital to life, but patients with systemic disorder such as RAS-associated lymphoproliferative disorder (RALD) can be maintained on thalidomide/sirolimus, which act downstream/parallel to this pathway. Thus, if a therapy, which aims in blocking a vitally important node(s), is likely to be associated with lethal sides effects, a downstream inhibition of the pathway should be the approach.

Finally, the reviewer recommends increasing the number of cancer networks analyzed for our study. Unfortunately, there is a scarcity of well-curated, trustworthy data that can be used for this purpose and we analyze all of the networks available in KEGG. While there may be other institutional or laboratory based data sets, they are not available to us. We have used all the KEGG networks for which good statistical data could be obtained.

### Reviewer 3: Dr. Marek Kimmel Rice University, United States of America

Application of gene and protein networks is likely to help tackle various issues in molecular evolution of cancers and from this viewpoint this is an interesting paper.

My criticism of the paper has more to do with the way the authors interpret data.

1. Tumor stratification by stage at diagnosis and cell type. Survival in most tumors is correlated inversely with stage at diagnosis. If deregulation of gene or protein network also depends on stage (quite likely), this will create association even if causality is absent. Therefore, stratification by stage is so important in cancer studies. SEER database includes stage information for major cancers such as for example lung cancer. In my opinion, the data analysis section should be supplemented by stratified analyses.
2. It is known that given sufficiently many parameters (or for example, many possible arrangements of mutations over the nodes of a network) it is possible to find some that correlate with outcome such as survival. Therefore, it is useful to randomly split the data set into test and validation samples. If the test developed works satisfactorily in the validation sample, then this makes it plausible. SEER or another database will be helpful for this purpose.
3. Relationship in Fig. 2 looks much more parabolic than linear, and the interpretation of Betti index value will be quite different in this latter case.

#### Author’s response to reviewer number 3

1. We agree with the reviewer, that analysis by stage at diagnosis and cell type would have been of interest. However, even though SEER provides stage-survival data, KEGG does not. The KEGG PPI networks are an average of many known networks for each specific cancer. We discuss the characteristics and construction of KEGG networks in our response to Reviewer 1. While, as the reviewer points out survival in most tumors is correlated inversely with stage at diagnosis, and while it is likely that deregulation of gene or protein network also depends on stage, the intention was not to create associations, but rather create an understanding about PPI networks and how to measure changes occurring with removal of a node. While stratification by stage is very important in cancers, stratified analyses may be difficult using present databases.
2. We understand the concept of leave-one/-many-out for cross validation. This is commonly done with machine learning algorithms. However, this was not a machine learning algorithm or algorithm using any kind of mapping. The method used here was straightforward evaluation of the relationship between a complexity measure of a PPI network and survival. Furthermore, KEGG does not provide any mutation information and the effect of mutation on networks could not be evaluated with KEGG. The individual cancer networks, as provided, are fixed averages across the number of subjects interrogated for the data.
3. We agree with the reviewer that a parabola could have been fitted to the sample set of cancers available in KEGG. It is likely, however, that this observational artifact is due to the paucity of information of more cancer types. As more data emerges, perhaps the study could be redone, and confirmed. Furthermore, fitting a parabola would be less helpful as we would have two sets of survival numbers for each Betti number. We do not advocate indiscriminately accepting the Betti computations, we provide a recommendation of an approach that can be further studied and/or combined with other methods of network interrogation.

#### Author’s response to second review by reviewer 1: Dr. Nathan J.Bowen (nominated by Dr. I.King Jordan), Clark Atlanta University

I would just like to point out that all KEGG pathway edges are not meant to represent protein-protein interactions. Many, for transcription factors, actually represent protein-DNA interactions. That is, the tf binds to the DNA in the promoter of the next node in the pathway. Also, the KEGG pathways do include mutation data. Both of these facts can be seen in the pathway for AML that the authors use, perhaps incorrectly, in their manuscript. The retinoic acid receptor, alpha (RARA) in Fig. 3 is one such transcription factor that regulates CCNA1 and JUP. The RARA in Fig. 3 is connected with an edge directly to CCNA and JUP even in the absence of direct protein-protein interaction. Likewise, the RARA in AML is depicted as two fusion genes in the KEGG pathways, PML-RARA and PLZF-RARA. The authors fail to include this in their pathways as well. This makes one question whether the authors took into account the regulatory interactions, that is, protein-DNA interactions that are depicted as edges in the KEGG pathways and maintained the fusion or other mutation data included from KEGG. In addition, the NFKB node that is removed in Pancreatic Cancer is a transcription factor and thus would only connect to DNA from one side in said network. For these reasons, I believe the authors should publish the tab delimited text files of the interaction networks used, so one can see how many transcription factor to target genes were included as protein-protein interactions. Likewise, the authors may wish to examine their title in which they explicitly reference protein-protein interactions.

#### Author’s response to the second review by Reviewer 1 Dr. Nathan J.Bowen

The KEGG networks are available from the KEGG website (www.KEGG.jp) and we now include images of the website as Additional file 1: Figure S1. As the reviewer points out, even though the KEGG maps consist not only of proteins but also of RNA, DNA and small molecules, and even though some of the interactions may represent protein-DNA interactions we do not focus on these. We have, for the purposes of this manuscript, focused on the protein-protein interaction network as generated by R-script KEGGgraph (described in the methods section). We also do not elaborate on mutation data, because mutation data are not embedded in the KEGG network, but rather link to these networks. The reviewer’s example of the PML-RARA interaction is not part of the main KEGG interaction map because these are abstracted away by KEGGgraph. The real human biology is immensely complex and we are in the early stages of accumulating the content of gene-gene, protein-protein or protein-gene interaction networks. While we share the reviewer’s concern about the incompleteness of these data sets, the mutation and fusion information is not produced in KEGGgraph operation for analysis. As requested, we now include the adjacency lists of the protein-protein interaction networks in Additional file 2: Table S1. We carefully considered the suggestion to reference other interactions in the title, but had concerns it may be misleading since our calculations are limited to protein-protein interactions.

## Additional files

Additional file 1: Figure S1. Diagrams of the Kyoto Encyclopedia of Genes and Genomes (KEGG) pathways for 11 cancers. The symbols used in the diagrams are summarized on the first page, and each specific cancer type is on subsequent pages in order of acute myeloid leukemia, bladder carcinoma, chronic myeloid leukemia, colorectal cancer, endometrial carcinoma, glioma, nonsmall-cell lung carcinoma, pancreatic carcinoma, renal cell carcinoma, small cell lung cancer, and thyroid cancer. All diagrams were downloaded from the KEGG PATHWAY Database (http://www.genome.jp/kegg/pathway.html). Additional file 2: Table S1. Adjacency Lists of proteins comprising the protein-protein interaction networks. It provides the EntrezID® for each protein and the respective gene symbols.

## Abbreviations

KEGG: Kyoto encyclopedia of genes and genomes
PPI: Protein-protein interaction network SEER, Surveillance epidemiology and end results database
AML: Acute myeloid leukemia
CML: Chronic myeloid leukemia
NSCL: Non-small cell lung carcinoma
SCL: Small cell lung carcinoma
