# Supplementary material for: Design principles for cancer therapy guided by changes in complexity of protein-protein interaction networks
Source: Biol Direct. 2015 May 28;10:32. doi: 10.1186/s13062-015-0058-5 (PMC4445818; doi:10.1186/s13062-015-0058-5)
Supplement: Additional file 1: Figure S1. — Diagrams of the Kyoto Encyclopedia of Genes and Genomes (KEGG) pathways for 11 cancers. The symbols used in the diagrams are summarized on the first page, and each specific cancer type is on subsequent pages in order of acute myeloid leukemia, bladder carcinoma, chronic myeloid leukemia, colorectal cancer, endometrial carcinoma, glioma, nonsmall-cell lung carcinoma, pancreatic carcinoma, renal cell carcinoma, small cell lung cancer, and thyroid cancer. All diagrams were downloaded from the KEGG PATHWAY Database (http://www.genome.jp/kegg/pathway.html). [file 13062_2015_58_MOESM1_ESM.pdf]

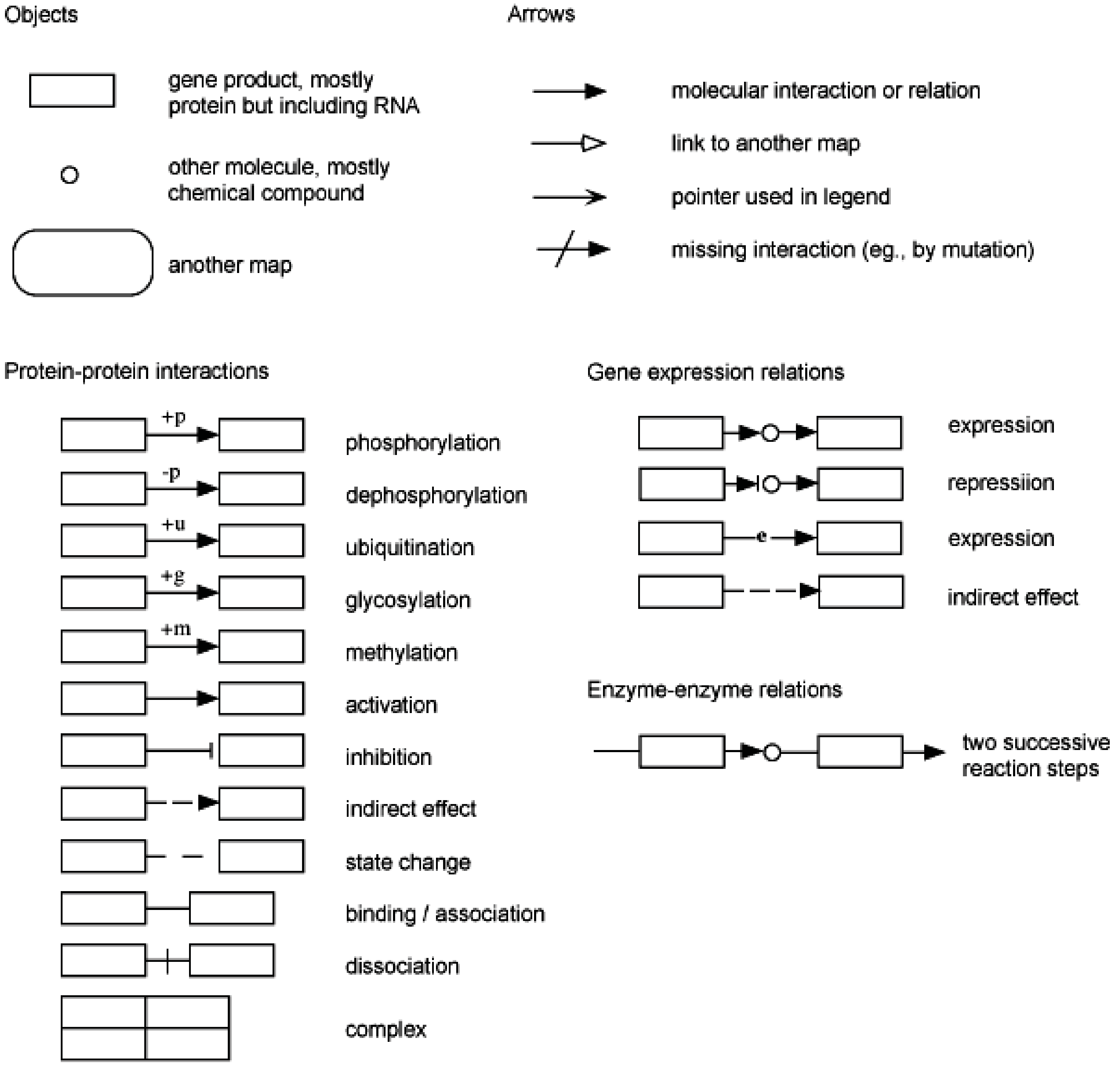
Objects
gene product, mostly protein but including RNA
other molecule, mostly chemical compound
another map
Arrows
molecular interaction or relation
link to another map
pointer used in legend
missing interaction (eg., by mutation)
Protein-protein interactions
+p
phosphorylation
-p
dephosphorylation
+u
ubiquitination
+g
glycosylation
+m
methylation
activation
inhibition
indirect effect
state change
binding / association
dissociation
complex
Gene expression relations
expression
repressiion
e
expression
indirect effect
Enzyme-enzyme relations
two successive reaction steps

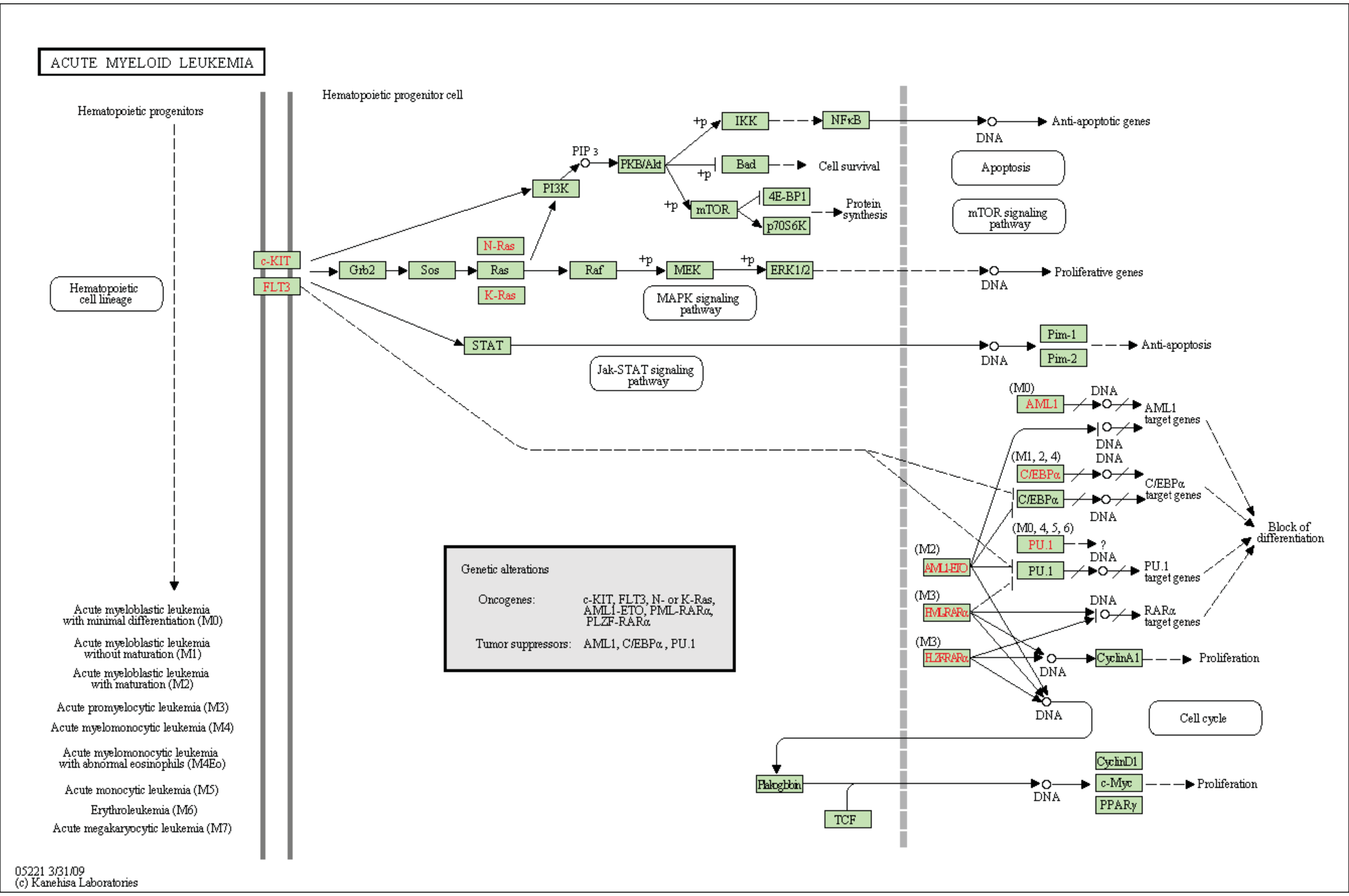
ACUTE MYELOID LEUKEMIA
Hematopoietic progenitors
Hematopoietic progenitor cell
Hematopoietic cell lineage
c-KIT
FLT3
Grb2
Sos
N-Ras
Ras
K-Ras
Raf
MEK
ERK1/2
+p
PI3K
PIP 3
PKB/Akt
IKK
NFκB
Bad
Cell survival
mTOR
4E-BP1
p70S6K
Protein synthesis
DNA
Anti-apoptotic genes
Apoptosis
mTOR signaling pathway
MAPK signaling pathway
Proliferative genes
STAT
Jak-STAT signaling pathway
Pim-1
Pim-2
Anti-apoptosis
(M0)
AML1
AML1 target genes
(M1, 2, 4)
C/EBPα
C/EBPα target genes
(M0, 4, 5, 6)
PU.1
?
PU.1 target genes
(M2)
AML1-ETO
(M3)
PML-RARα
RARα target genes
PLZF-RARα
CyclinA1
Proliferation
Block of differentiation
Cell cycle
Plakoglobin
TCF
CyclinD1
c-Myc
PPARγ
Genetic alterations
Oncogenes: c-KIT, FLT3, N- or K-Ras, AML1-ETO, PML-RARα, PLZF-RARα
Tumor suppressors: AML1, C/EBPα, PU.1
Acute myeloblastic leukemia with minimal differentiation (M0)
Acute myeloblastic leukemia without maturation (M1)
Acute myeloblastic leukemia with maturation (M2)
Acute promyelocytic leukemia (M3)
Acute myelomonocytic leukemia (M4)
Acute myelomonocytic leukemia with abnormal eosinophils (M4Eo)
Acute monocytic leukemia (M5)
Erythroleukemia (M6)
Acute megakaryocytic leukemia (M7)
05221 3/31/09
(c) Kanehisa Laboratories

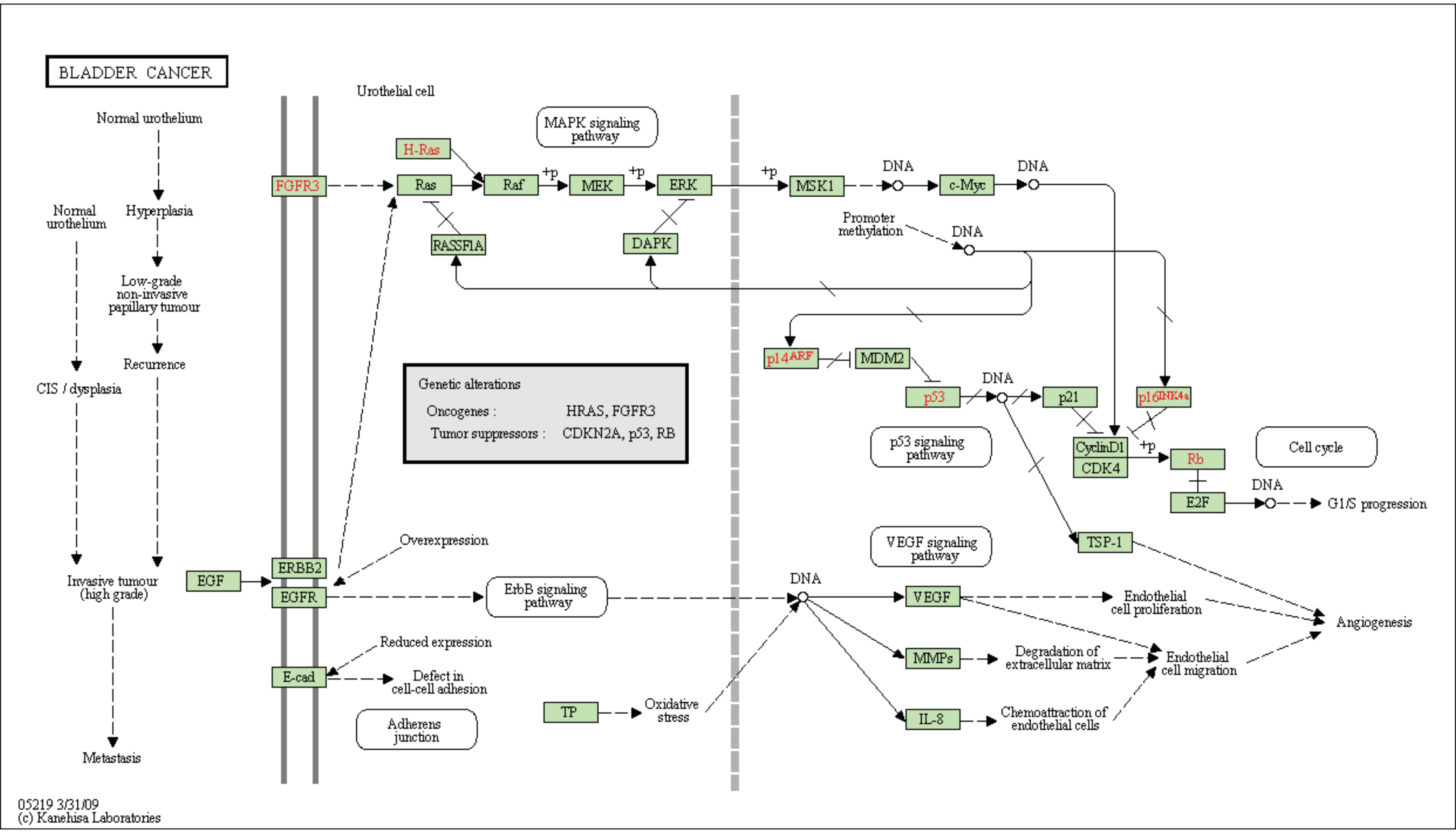
BLADDER CANCER
Urothelial cell
Normal urothelium
Hyperplasia
Normal urothelium
Low-grade non-invasive papillary tumour
Recurrence
CIS / dysplasia
Invasive tumour (high grade)
Metastasis
MAPK signaling pathway
H-Ras
FGFR3
Ras
Raf
MEK
ERK
+p
MSK1
DNA
c-Myc
RASSF1A
DAPK
Promoter methylation
p14ARF
MDM2
p53
p21
p16INK4a
CyclinD1
CDK4
Rb
E2F
G1/S progression
Cell cycle
p53 signaling pathway
Genetic alterations
Oncogenes : HRAS, FGFR3
Tumor suppressors : CDKN2A, p53, RB
VEGF signaling pathway
TSP-1
Overexpression
EGF
ERBB2
EGFR
ErbB signaling pathway
VEGF
Endothelial cell proliferation
Angiogenesis
MMPs
Degradation of extracellular matrix
Endothelial cell migration
IL-8
Chemoattraction of endothelial cells
Reduced expression
E-cad
Defect in cell-cell adhesion
Adherens junction
TP
Oxidative stress
05219 3/31/09
(c) Kanehisa Laboratories

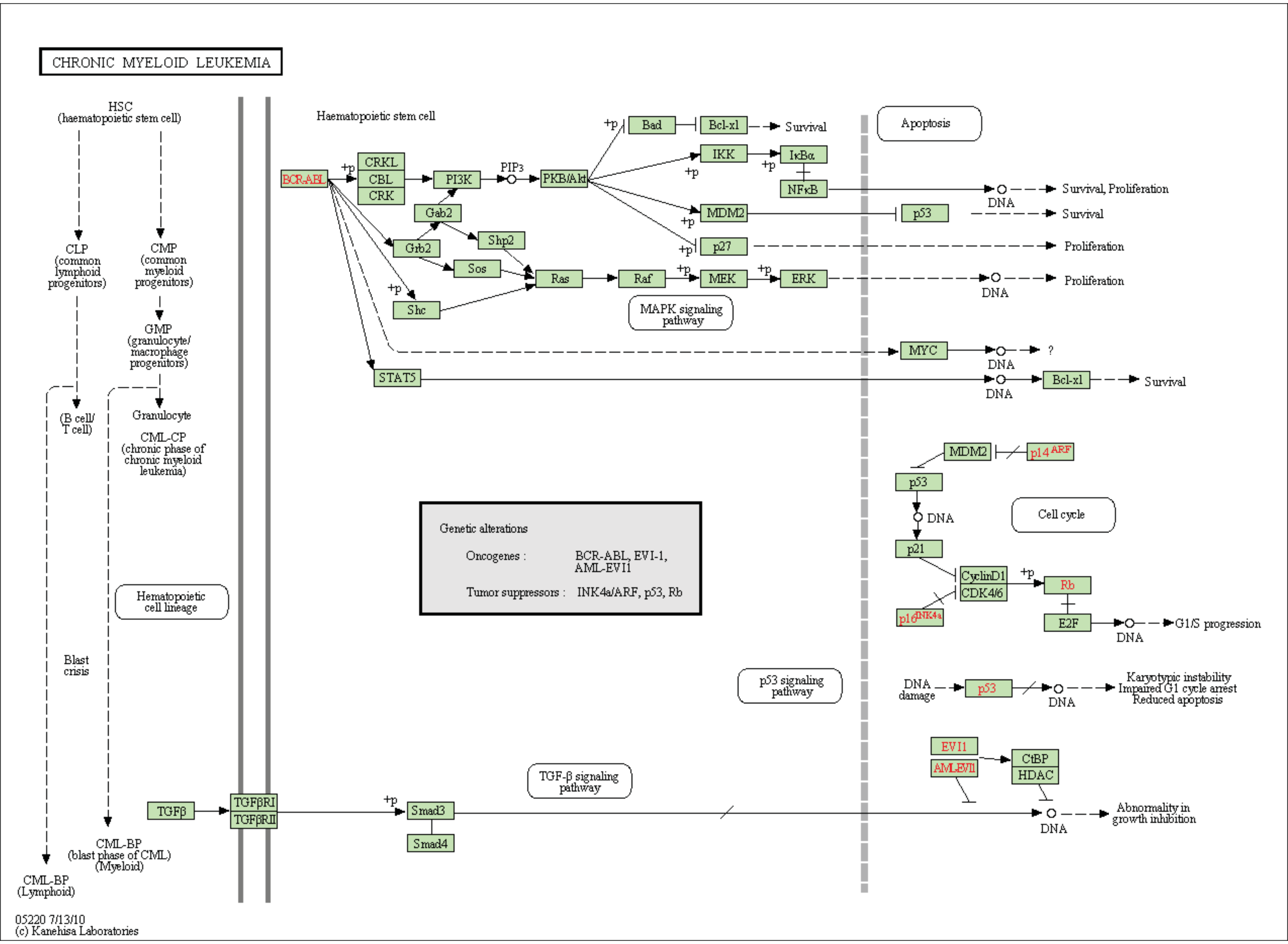

CHRONIC MYELOID LEUKEMIA
HSC
(haematopoietic stem cell)
CLP
(common lymphoid progenitors)
CMP
(common myeloid progenitors)
GMP
(granulocyte/ macrophage progenitors)
(B cell/ T cell)
Granulocyte
CML-CP
(chronic phase of chronic myeloid leukemia)
Hematopoietic cell lineage
Blast crisis
CML-BP
(blast phase of CML)
(Myeloid)
CML-BP
(Lymphoid)
Haematopoietic stem cell
BCR-ABL
+p
CRKL
CBL
CRK
PI3K
PIP3
PKB/Akt
Gab2
Grb2
Shp2
Sos
Shc
Ras
Raf
MEK
ERK
Bad
Bcl-xl
Survival
IKK
IκBα
NFκB
MDM2
p53
p27
Apoptosis
DNA
Survival, Proliferation
Survival
Proliferation
Proliferation
MAPK signaling pathway
MYC
?
STAT5
Bcl-xl
Survival
MDM2
p14 ARF
p53
DNA
p21
Cell cycle
CyclinD1
CDK4/6
Rb
p16INK4a
E2F
G1/S progression
Genetic alterations
Oncogenes : BCR-ABL, EVI-1, AML-EVI1
Tumor suppressors : INK4a/ARF, p53, Rb
p53 signaling pathway
DNA damage
p53
Karyotypic instability
Impaired G1 cycle arrest
Reduced apoptosis
EVI1
AML-EVI1
CtBP
HDAC
TGF-β signaling pathway
TGFβ
TGFβRI
TGFβRII
Smad3
Smad4
Abnormality in growth inhibition
05220 7/13/10
(c) Kanehisa Laboratories

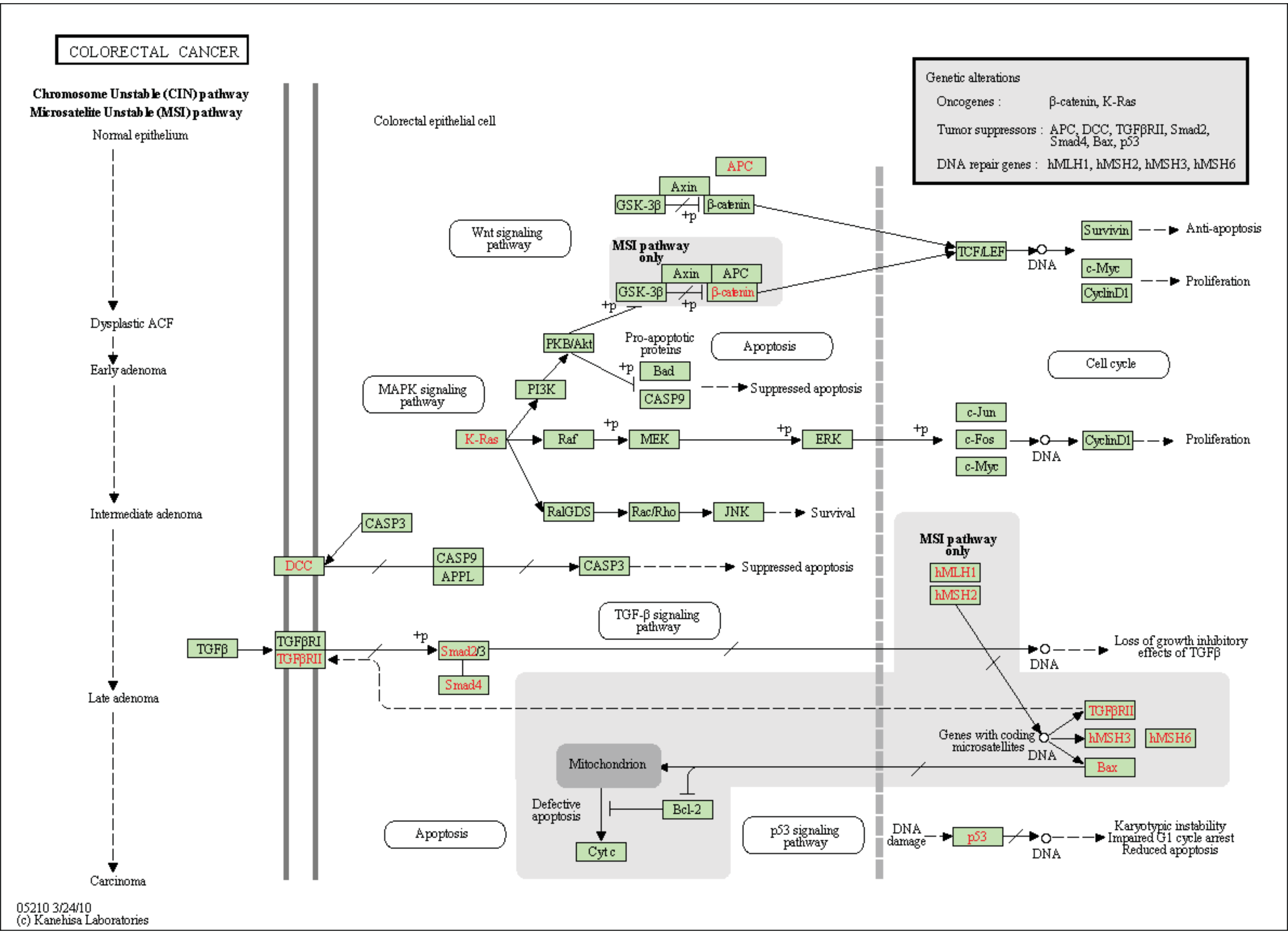
COLORECTAL CANCER
Chromosome Unstable (CIN) pathway
Microsatellite Unstable (MSI) pathway
Normal epithelium
Dysplastic ACF
Early adenoma
Intermediate adenoma
Late adenoma
Carcinoma
Colorectal epithelial cell
Genetic alterations
Oncogenes : β-catenin, K-Ras
Tumor suppressors : APC, DCC, TGFβRII, Smad2, Smad4, Bax, p53
DNA repair genes : hMLH1, hMSH2, hMSH3, hMSH6
APC
Axin
GSK-3β
β-catenin
+p
Wnt signaling pathway
MSI pathway only
Axin
APC
GSK-3β
β-catenin
+p
TCF/LEF
DNA
Survivin
Anti-apoptosis
c-Myc
CyclinD1
Proliferation
PKB/Akt
Pro-apoptotic proteins
Bad
CASP9
Suppressed apoptosis
Apoptosis
Cell cycle
MAPK signaling pathway
PI3K
K-Ras
Raf
MEK
ERK
c-Jun
c-Fos
c-Myc
CyclinD1
Proliferation
RalGDS
Rac/Rho
JNK
Survival
CASP3
DCC
CASP9
APPL
CASP3
Suppressed apoptosis
MSI pathway only
hMLH1
hMSH2
TGFβ
TGFβRI
TGFβRII
Smad2/3
Smad4
TGF-β signaling pathway
Loss of growth inhibitory effects of TGFβ
TGFβRII
Genes with coding microsatellites
hMSH3
hMSH6
Bax
Mitochondrion
Defective apoptosis
Bcl-2
Cyt c
Apoptosis
p53 signaling pathway
DNA damage
p53
Karyotypic instability
Impaired G1 cycle arrest
Reduced apoptosis
05210 3/24/10
(c) Kanehisa Laboratories

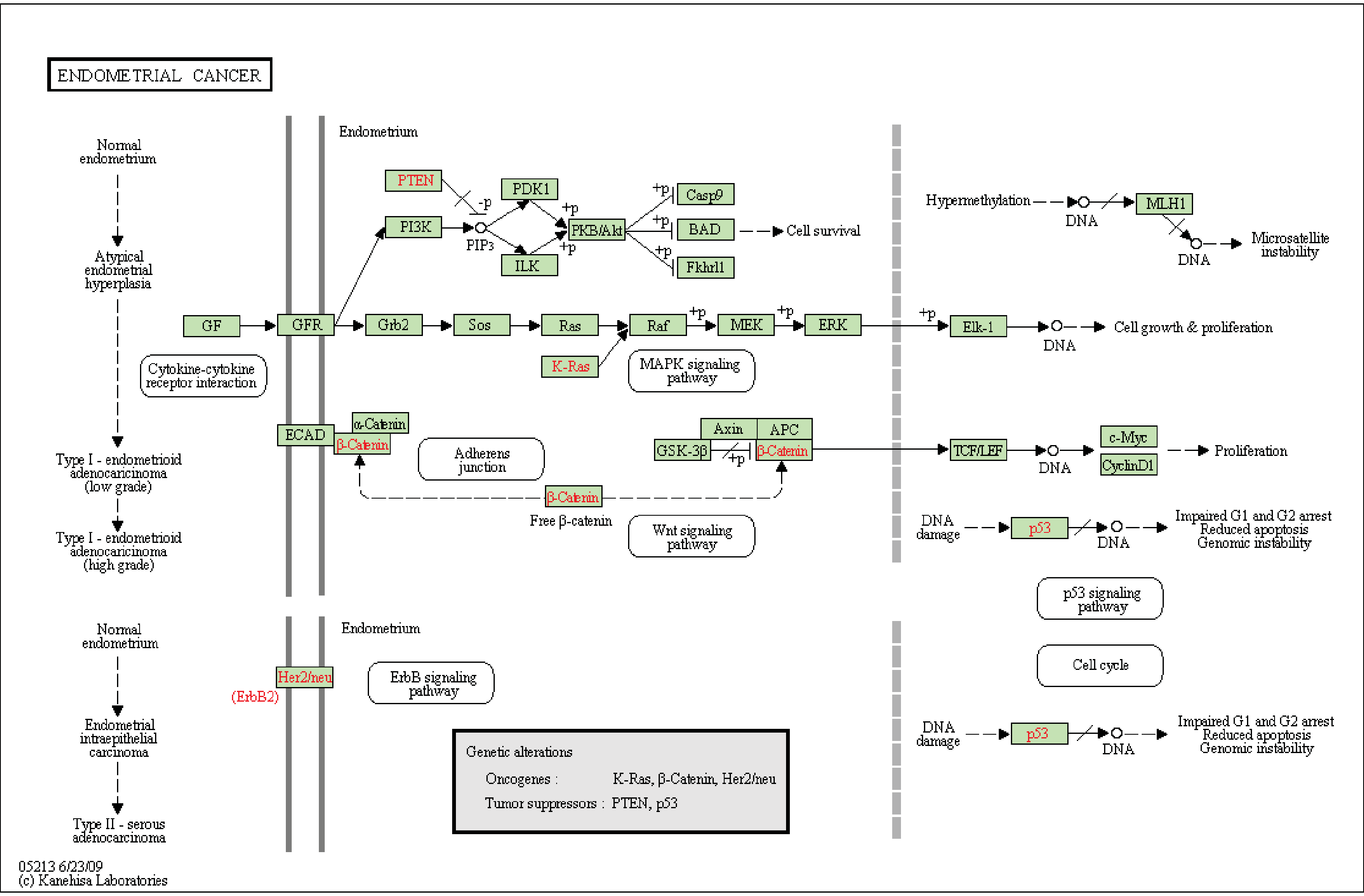

ENDOMETRIAL CANCER
Normal endometrium
Atypical endometrial hyperplasia
Type I - endometrioid adenocarcinoma (low grade)
Type I - endometrioid adenocarcinoma (high grade)
Endometrium
PTEN
PI3K
PIP3
PDK1
ILK
PKB/Akt
Casp9
BAD
Fkhrl1
Cell survival
GF
GFR
Grb2
Sos
Ras
Raf
MEK
ERK
K-Ras
MAPK signaling pathway
Cytokine-cytokine receptor interaction
Elk-1
DNA
Cell growth & proliferation
Hypermethylation
MLH1
Microsatellite instability
ECAD
α-Catenin
β-Catenin
Adherens junction
Axin
APC
GSK-3β
TCF/LEF
c-Myc
CyclinD1
Proliferation
Free β-catenin
Wnt signaling pathway
DNA damage
p53
Impaired G1 and G2 arrest
Reduced apoptosis
Genomic instability
p53 signaling pathway
Cell cycle
Normal endometrium
Endometrial intraepithelial carcinoma
Type II - serous adenocarcinoma
Her2/neu
(ErbB2)
ErbB signaling pathway
Genetic alterations
Oncogenes : K-Ras, β-Catenin, Her2/neu
Tumor suppressors : PTEN, p53
05213 6/23/09
(c) Kanehisa Laboratories

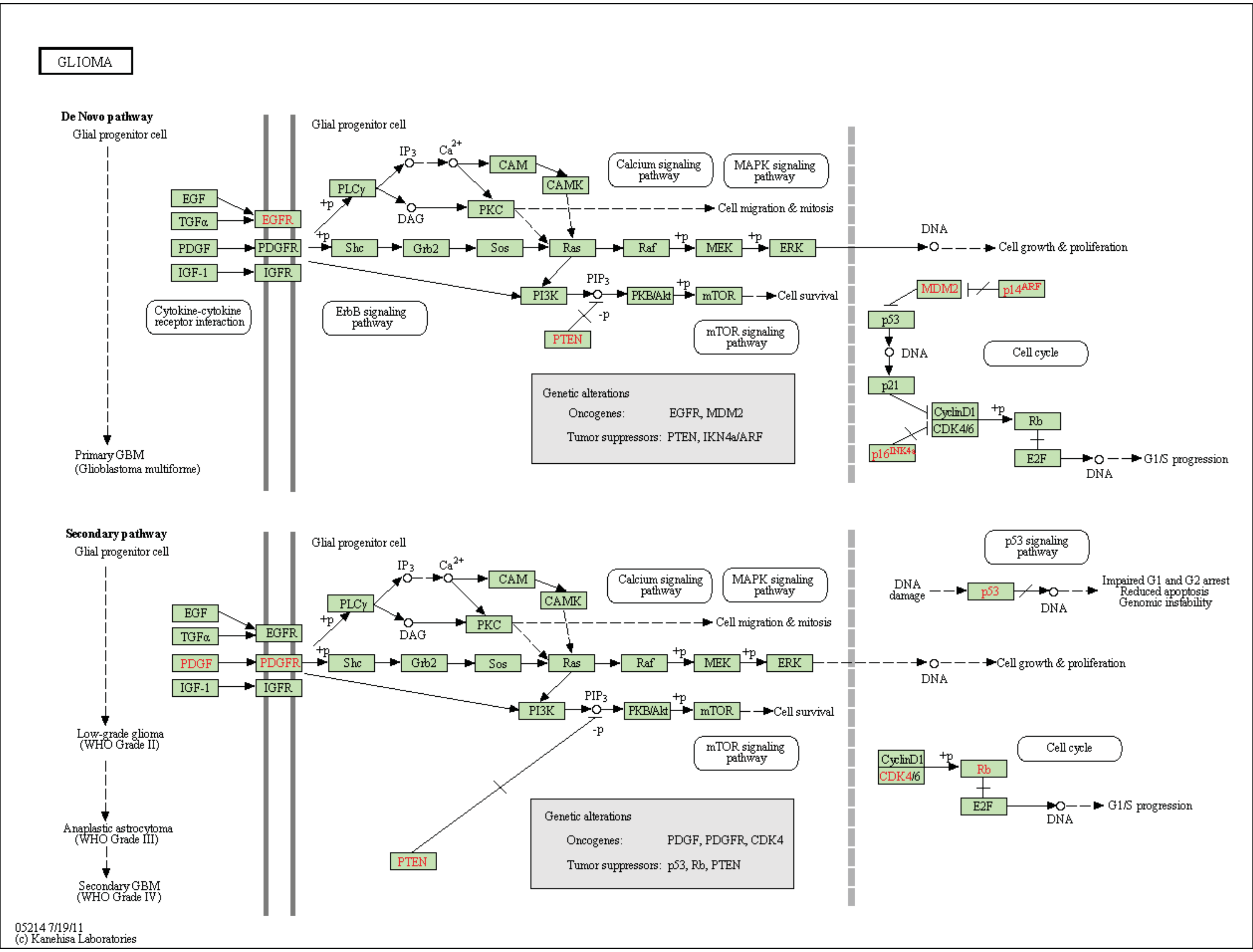
GLIOMA
De Novo pathway
Glial progenitor cell
Primary GBM
(Glioblastoma multiforme)
EGF
TGFα
PDGF
IGF-1
EGFR
PDGFR
IGFR
Cytokine-cytokine receptor interaction
Glial progenitor cell
PLCγ
IP3
Ca2+
CAM
CAMK
DAG
PKC
Shc
Grb2
Sos
Ras
Raf
MEK
ERK
PI3K
PIP3
PKB/Akt
mTOR
PTEN
Calcium signaling pathway
MAPK signaling pathway
ErbB signaling pathway
mTOR signaling pathway
Cell migration & mitosis
Cell survival
DNA
Cell growth & proliferation
MDM2
p14ARF
p53
p21
CyclinD1
CDK4/6
p16INK4a
Rb
E2F
G1/S progression
Cell cycle
Genetic alterations
Oncogenes: EGFR, MDM2
Tumor suppressors: PTEN, IKN4a/ARF
Secondary pathway
Glial progenitor cell
Low-grade glioma
(WHO Grade II)
Anaplastic astrocytoma
(WHO Grade III)
Secondary GBM
(WHO Grade IV)
p53 signaling pathway
DNA damage
Impaired G1 and G2 arrest
Reduced apoptosis
Genomic instability
Genetic alterations
Oncogenes: PDGF, PDGFR, CDK4
Tumor suppressors: p53, Rb, PTEN
05214 7/19/11
(c) Kanehisa Laboratories

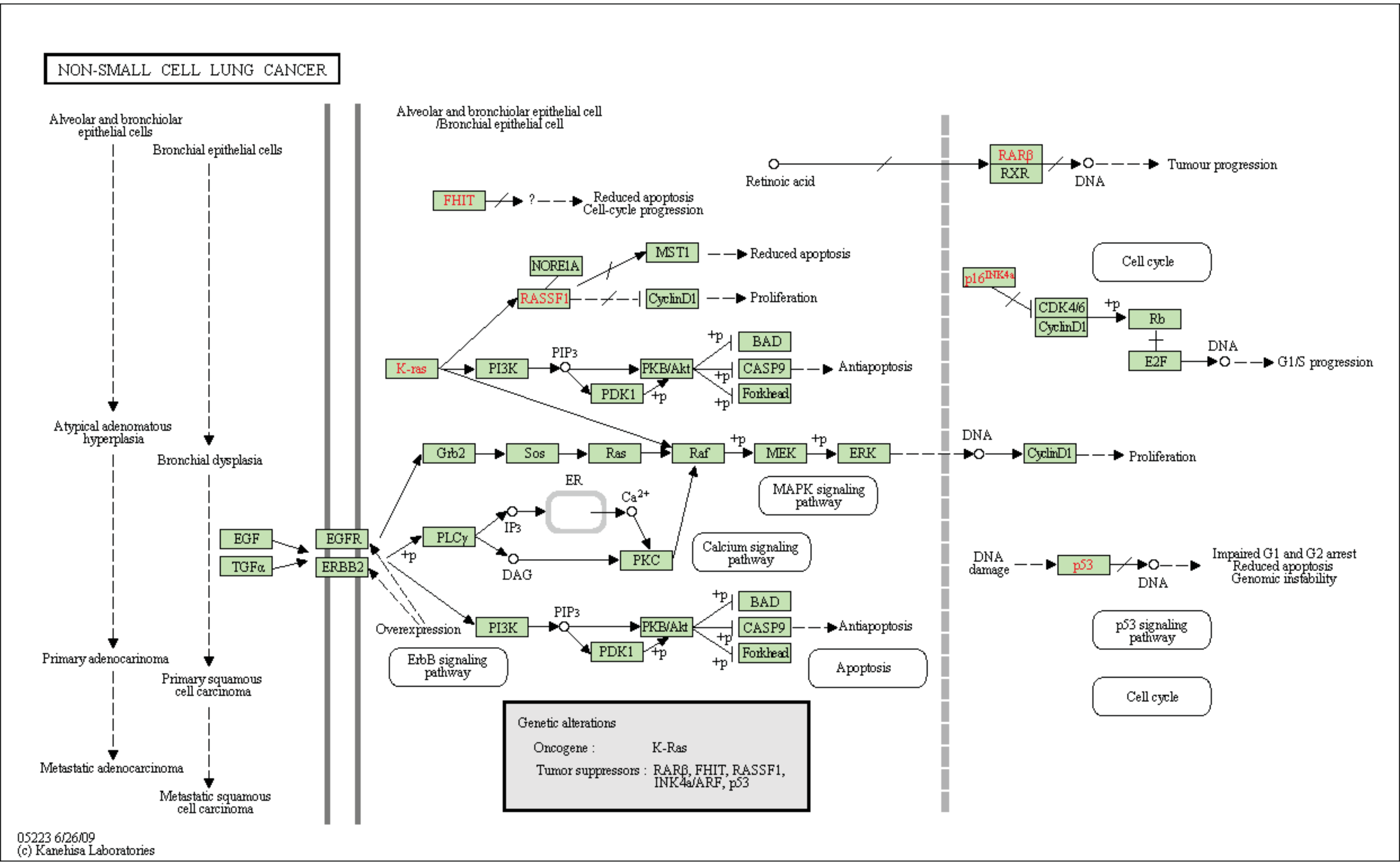
NON-SMALL CELL LUNG CANCER
Alveolar and bronchiolar epithelial cells
Bronchial epithelial cells
Atypical adenomatous hyperplasia
Bronchial dysplasia
Primary adenocarinoma
Primary squamous cell carcinoma
Metastatic adenocarcinoma
Metastatic squamous cell carcinoma
Alveolar and bronchiolar epithelial cell /Bronchial epithelial cell
FHIT
? Reduced apoptosis Cell-cycle progression
Retinoic acid
RARβ
RXR
DNA
Tumour progression
NORE1A
MST1
Reduced apoptosis
RASSF1
CyclinD1
Proliferation
p16INK4a
Cell cycle
CDK4/6
CyclinD1
+p
Rb
E2F
DNA
G1/S progression
K-ras
PI3K
PIP3
PKB/Akt
PDK1
+p
BAD
CASP9
Forkhead
Antiapoptosis
Grb2
Sos
Ras
Raf
MEK
ERK
DNA
CyclinD1
Proliferation
MAPK signaling pathway
ER
Ca2+
IP3
DAG
PLCγ
PKC
Calcium signaling pathway
EGF
TGFα
EGFR
ERBB2
Overexpression
ErbB signaling pathway
Apoptosis
DNA damage
p53
DNA
Impaired G1 and G2 arrest
Reduced apoptosis
Genomic instability
p53 signaling pathway
Cell cycle
Genetic alterations
Oncogene : K-Ras
Tumor suppressors : RARβ, FHIT, RASSF1, INK4a/ARF, p53
05223 6/26/09
(c) Kanehisa Laboratories

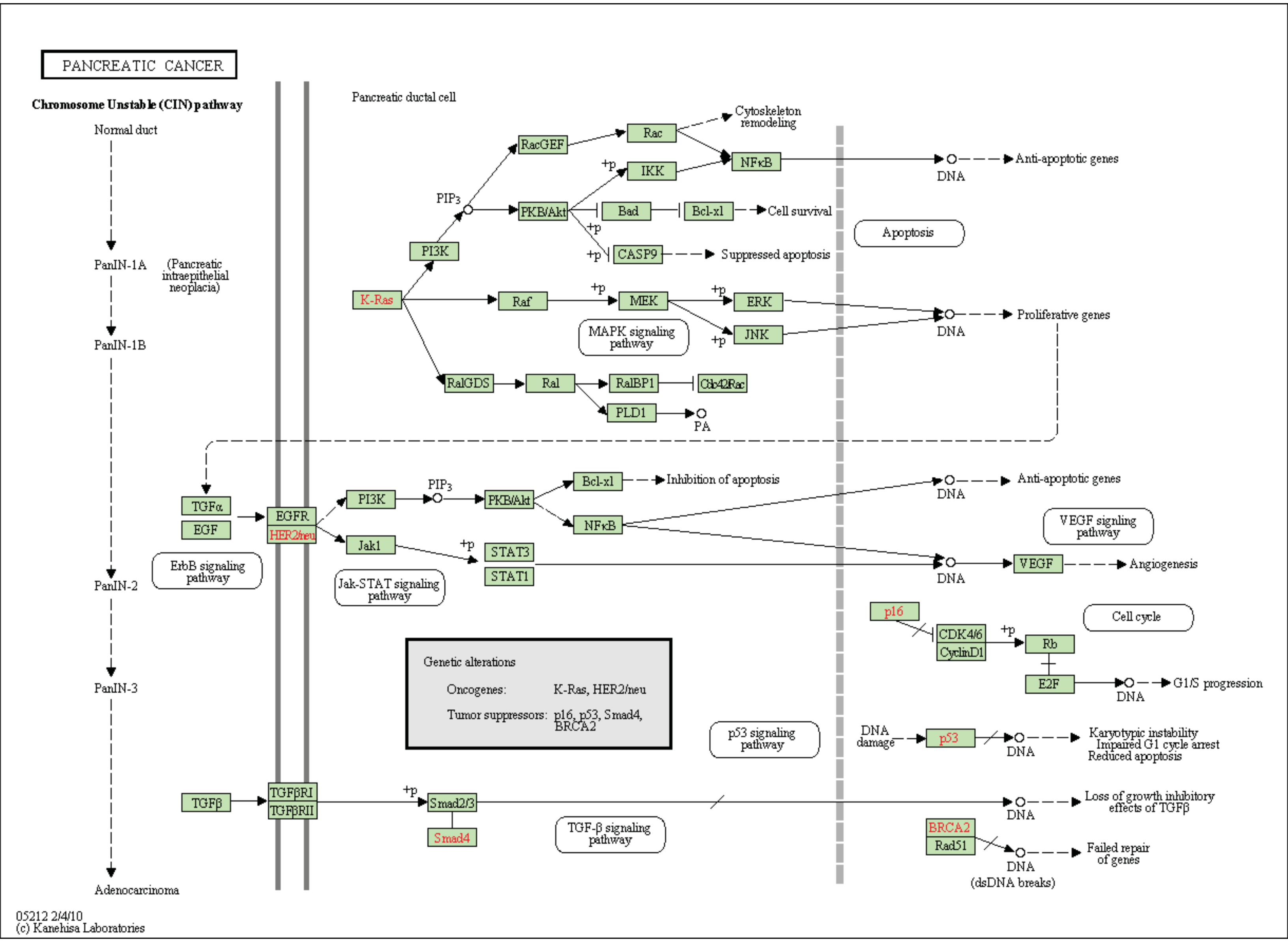
PANCREATIC CANCER
Chromosome Unstable (CIN) pathway
Normal duct
PanIN-1A
(Pancreatic intraepithelial neoplacia)
PanIN-1B
PanIN-2
PanIN-3
Adenocarcinoma
Pancreatic ductal cell
RacGEF
Rac
Cytoskeleton remodeling
NFκB
DNA
Anti-apoptotic genes
+p
IKK
PIP3
PKB/Akt
Bad
Bcl-xl
Cell survival
Apoptosis
PI3K
CASP9
Suppressed apoptosis
K-Ras
Raf
MEK
ERK
JNK
Proliferative genes
MAPK signaling pathway
RalGDS
Ral
RalBP1
Cdc42/Rac
PLD1
PA
TGFα
EGF
EGFR
HER2/neu
ErbB signaling pathway
PI3K
PIP3
PKB/Akt
Bcl-xl
Inhibition of apoptosis
NFκB
Anti-apoptotic genes
VEGF signaling pathway
Jak1
STAT3
STAT1
Jak-STAT signaling pathway
VEGF
Angiogenesis
p16
CDK4/6
CyclinD1
Rb
Cell cycle
E2F
G1/S progression
Genetic alterations
Oncogenes: K-Ras, HER2/neu
Tumor suppressors: p16, p53, Smad4, BRCA2
p53 signaling pathway
DNA damage
p53
Karyotypic instability
Impaired G1 cycle arrest
Reduced apoptosis
TGFβ
TGFβRI
TGFβRII
Smad2/3
Smad4
TGF-β signaling pathway
Loss of growth inhibitory effects of TGFβ
BRCA2
Rad51
Failed repair of genes
(dsDNA breaks)
05212 2/4/10
(c) Kanehisa Laboratories

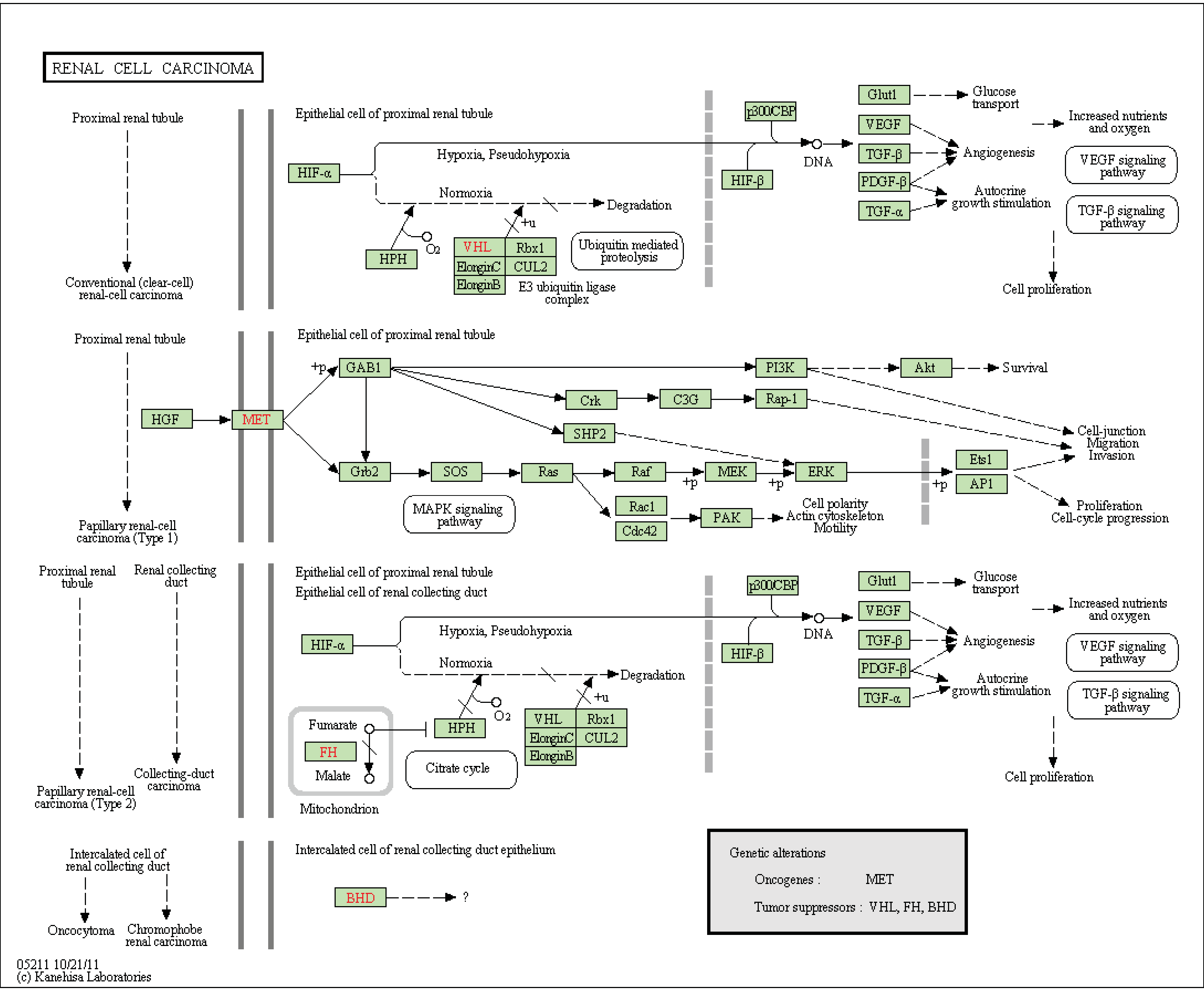

RENAL CELL CARCINOMA
Proximal renal tubule
Conventional (clear-cell) renal-cell carcinoma
Epithelial cell of proximal renal tubule
HIF-α
Hypoxia, Pseudohypoxia
Normoxia
Degradation
HPH
O2
VHL
Rbx1
ElonginC
CUL2
ElonginB
+u
E3 ubiquitin ligase complex
Ubiquitin mediated proteolysis
p300/CBP
HIF-β
DNA
Glut1
VEGF
TGF-β
PDGF-β
TGF-α
Glucose transport
Angiogenesis
Autocrine growth stimulation
Increased nutrients and oxygen
VEGF signaling pathway
TGF-β signaling pathway
Cell proliferation
Proximal renal tubule
Papillary renal-cell carcinoma (Type 1)
Epithelial cell of proximal renal tubule
HGF
MET
+p
GAB1
Grb2
SOS
Ras
Raf
MEK
ERK
PI3K
Akt
Survival
Crk
C3G
Rap-1
SHP2
Rac1
Cdc42
PAK
Cell polarity Actin cytoskeleton Motility
MAPK signaling pathway
Ets1
AP1
Cell-junction Migration Invasion
Proliferation Cell-cycle progression
Proximal renal tubule
Renal collecting duct
Papillary renal-cell carcinoma (Type 2)
Collecting-duct carcinoma
Epithelial cell of proximal renal tubule
Epithelial cell of renal collecting duct
Fumarate
FH
Malate
Mitochondrion
Citrate cycle
Intercalated cell of renal collecting duct
Oncocytoma
Chromophobe renal carcinoma
Intercalated cell of renal collecting duct epithelium
BHD
?
Genetic alterations
Oncogenes : MET
Tumor suppressors : VHL, FH, BHD
05211 10/21/11
(c) Kanehisa Laboratories

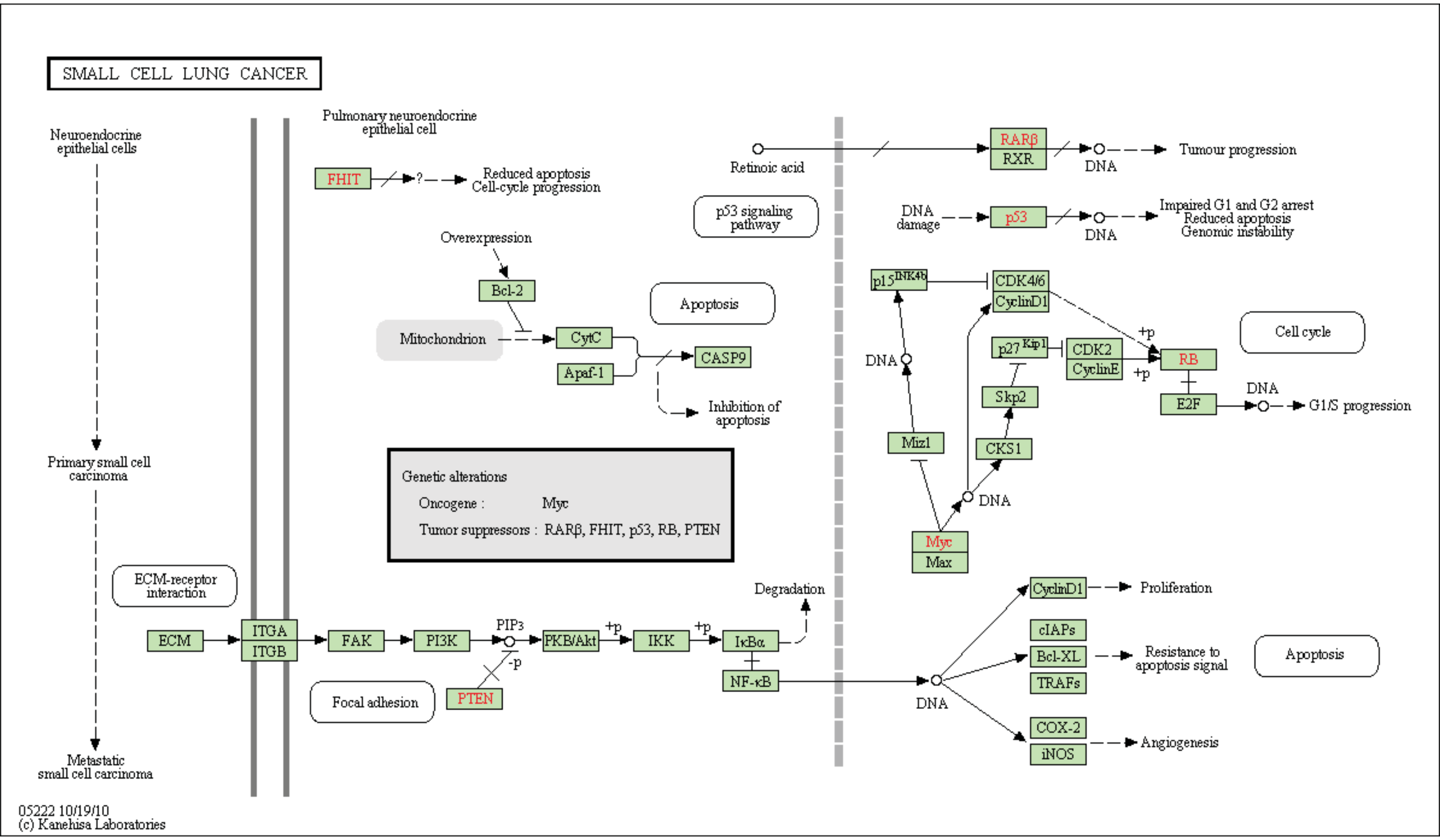

SMALL CELL LUNG CANCER
Neuroendocrine epithelial cells
Primary small cell carcinoma
Metastatic small cell carcinoma
Pulmonary neuroendocrine epithelial cell
FHIT
?
Reduced apoptosis
Cell-cycle progression
p53 signaling pathway
Overexpression
Bcl-2
Apoptosis
Mitochondrion
CytC
Apaf-1
CASP9
Inhibition of apoptosis
Genetic alterations
Oncogene : Myc
Tumor suppressors : RARβ, FHIT, p53, RB, PTEN
ECM-receptor interaction
ECM
ITGA
ITGB
FAK
PI3K
PIP3
-p
PTEN
PKB/Akt
+p
IKK
+p
IκBα
NF-κB
Degradation
Focal adhesion
Retinoic acid
RARβ
RXR
DNA
Tumour progression
DNA damage
p53
DNA
Impaired G1 and G2 arrest
Reduced apoptosis
Genomic instability
p15INK4b
CDK4/6
CyclinD1
+p
RB
Cell cycle
DNA
p27 Kip1
CDK2
CyclinE
+p
Skp2
E2F
DNA
G1/S progression
Miz1
CKS1
DNA
Myc
Max
CyclinD1
Proliferation
cIAPs
Bcl-XL
TRAFs
Resistance to apoptosis signal
Apoptosis
DNA
COX-2
iNOS
Angiogenesis
05222 10/19/10
(c) Kanehisa Laboratories

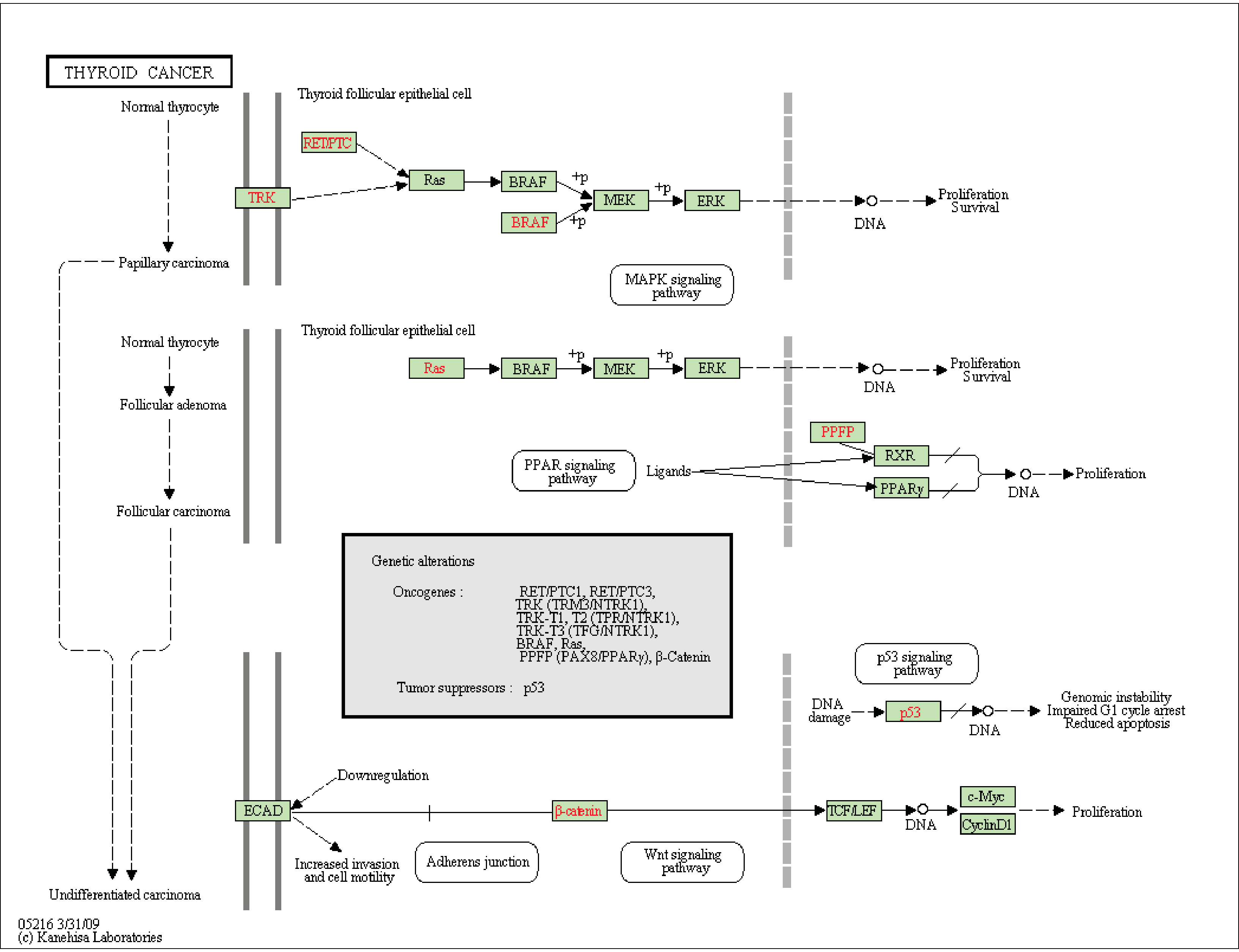
THYROID CANCER
Normal thyrocyte
Thyroid follicular epithelial cell
RET/PTC
TRK
Ras
BRAF
BRAF
+p
+p
MEK
+p
ERK
DNA
Proliferation
Survival
Papillary carcinoma
MAPK signaling pathway
Normal thyrocyte
Thyroid follicular epithelial cell
Ras
BRAF
+p
MEK
+p
ERK
DNA
Proliferation
Survival
Follicular adenoma
PPFP
RXR
PPAR signaling pathway
Ligands
PPARγ
DNA
Proliferation
Follicular carcinoma
Genetic alterations
Oncogenes :
RET/PTC1, RET/PTC3,
TRK (TRM3/NTRK1),
TRK-T1, T2 (TPR/NTRK1),
TRK-T3 (TFG/NTRK1),
BRAF, Ras,
PPFP (PAX8/PPARγ), β-Catenin
Tumor suppressors : p53
p53 signaling pathway
DNA damage
p53
DNA
Genomic instability
Impaired G1 cycle arrest
Reduced apoptosis
Downregulation
ECAD
β-catenin
TCF/LEF
DNA
c-Myc
CyclinD1
Proliferation
Increased invasion and cell motility
Adherens junction
Wnt signaling pathway
Undifferentiated carcinoma
05216 3/31/09
(c) Kanehisa Laboratories
